# EphB4-ephrin-B2 are targets in castration resistant prostate cancer

**DOI:** 10.1038/s41416-025-02942-5

**Published:** 2025-03-05

**Authors:** Grace Xiuqing Li, Binyun Ma, Shaobing Zhang, Ren Liu, Imran N. Siddiqi, Akash Sali, Anthony El-Khoueiry, Mitchell Gross, Bodour Salhia, Sarmad Sadeghi, Parkash S. Gill

**Affiliations:** 1 Department of Medicine, USC Norris Comprehensive Cancer Center, Keck School of Medicine, University of Southern California, Los Angeles, CA USA; 2 Merck Pharmaceutical Inc. Previous: Department of Medicine, USC Norris Comprehensive Cancer Center, Keck School of Medicine, University of Southern California, Los Angeles, CA USA; 3 Department of Pathology, USC Norris Comprehensive Cancer Center, Keck School of Medicine, University of Southern California, Los Angeles, CA USA; 4 Catherine & Joseph Aresty Department of Urology, USC Institute of Urology, Keck School of Medicine, University of Southern California, Los Angeles, CA USA; 5 Department of Translational Genomics, Keck School of Medicine, University of Southern California, Los Angeles, CA USA

**Keywords:** Prostate cancer, Target identification

## Abstract

**Background:**

PI3K pathway activation is a common and early event in prostate cancer, from loss of function mutations in *PTEN*, or activating mutations in PIK3Ca or AKT leading to constitutive activation, induction of growth factor-receptors kinase EphB4 and its ligand ephrin-B2. We hypothesized that induction of EphB4 is an early event required for tumor initiation. Secondly, we hypothesized that EphB4 remains relevant when prostate cancer becomes androgen independent.

**Methods:**

Genetic mouse model of conditional *PTEN* deletion in prostate epithelium induces tumor in all mice. We tested this model against *EPHB4* wild type and deleted in prostate epithelium. This allowed us to test its role in tumor initiation. We also tested an orthogonal approach by using decoy soluble EphB4 to block bidirectional signaling resulting from EphB4-ephrin-B2 interaction. Role of EphB4-ephrin-B2 in androgen deprived mice was tested for role in refractory cancer model.

**Results:**

*PTEN* deletion induces EphB4 and ephrin-B2 in prostate cancer which was substantially reduced when *EPHB4* is deleted in the same prostate epithelial cells. sEphB4-alb fusion protein with improved pharmacokinetics similarly inhibited tumor formation, thus establishing the role in tumor initiation. sEphB4-alb retained the efficacy in castration resistant androgen independent prostate cancer. We have thus observed that induction of EphB4 is required for the initiation of prostate cancer in *PTEN* null mouse and that signaling downstream from EphB4 is required in androgen deprivation and thus castration resistant prostate cancer. Pharmacological inhibition of EphB4 pathway reproduced the results. Targeting EphB4 should be tested in prostate cancer especially those resistant to androgen deprivation therapy.

**Conclusions:**

EphB4 and ephrin-B2 receptor ligand pair is induced in *PTEN* null prostate cancer, which significantly contributes to the tumor initiation. Secondly, EphB4-ephrin-B2 pathway continue to promote tumor progression even in androgen deprivation and thus hormone refractory tumor. EphB4-ephrin-B2 may be candidates for precision medicine with biomarker-based patient selection with and without concurrent standard of care.

## Introduction

Prostate cancer is the most prevalent non-skin cancer in men and the second most common cause of death from cancer in men, resulting in around 30,000 deaths in the United States each year. In cases that cannot be cured by surgery or radiation, androgen deprivation therapy (ADT) is the primary mode of treatment, although it frequently fails when cancers escape from androgen dependence and progress to castration-resistant prostate cancers (CRPC). Next-generation sequencing has identified mutations in the phosphatidylinositol-4,5-bisphosphate 3-kinase (PI3K) pathway, including mutations in PI3KCA and phosphatase and tension homolog (PTEN) as early events in PC [1–3]. Consequently, PI3K isoform specific inhibitors are under investigation. Concurrent alterations in *AR, TP53, RB*, and *MYC* genes [4–6] in tumor resistant to androgen deprivation offer other opportunities to study tumor biology and targeted intervention. Identification of unique proteins on tumor cell surface offer other opportunities for intervention. Here, we describe the role of ephrin receptor EphB4 and its ligand ephrin-B2 in the development and progression of PC and CRPC.

Ephrin (Eph) receptors are the largest subgroup of the receptor tyrosine kinase (RTK) family [NCBI’s Reference Sequence (RefSeq) database]. Together with their ligands, the ephrins, Eph receptors regulate biological functions in cell localization, tissue boundary formation, axon guidance, positioning of precursor and mature cells in the intestinal crypt and villus cells [7], and urethral development [8–10]. Ephrins are divided into two groups: EphA (EphA1-8,10) subgroup ligands (ephrinA1-5) localize to the cell surface via glycosyl-phosphatidylinositol GPI anchors, while EphB (EphB1-4,6) family ligands (ephrinB1-3) are transmembrane proteins [11]. Eph-ephrin interactions occur upon cell-cell contact with dimers of Eph and Ephrin forming high affinity hetero-tetrameric complexes that trigger bidirectional signaling in receptor- and ligand-expressing cells [12, 13]. Each receptor and ligand within the subgroups can bind promiscuously [14], except for EphB4 which only binds ephrin-B2. EphB4 and its ligand ephrin-B2 are expressed in developing venous and arterial capillaries respectively in the embryo and are essential for the maturation of the vascular system. Knockout of either is embryonic lethal [15, 16]. However, their expression is markedly reduced in adult life with aberrant re-expression occurring in tumor vessels and tumor cells [17–19].

We and others have described elevated levels of EphB4 in prostate cancer [20–22] and shown that EphB4 is induced in prostate cancer by both the PI3K pathway and the β-catenin pathway [23, 24]. EphB4 provides a survival advantage in tumor cells by further activating the PI3K pathway [23], unlike EphB2, which is expressed in normal gland and functions as a tumor suppressor [7, 23, 25–28].

Downstream signaling also differs between EphB4 and EphB2, favoring invasive phenotype with EphB4 which activates CDC42, and overcomes cell contact inhibition [22] while EphB2 induces Rho-A small GTPase which leads to cell contact inhibition and cell retraction [29].

To test the contribution of EphB4 downstream from *PTEN* null prostate cancer, we developed conditional knock out of EphB4 and crossed with conditional *PTEN* null mice. We also tested pharmacological inhibitor of EphB4-ephrin-B2 pathway with decoy soluble EphB4-albumin fusion protein(sEphb4-alb) [30, 31]. Loss of EphB4 prevented the development of prostate cancer, and the results were replicated with sEphB4-alb. Furthermore, castration resistant tumor retains dependence on EphB4 as sEphB4-alb induced remission, concurrent with decrease in PI3K signaling and Myc protein levels.

## Methods

### *PTEN*-null prostate cancer mouse model

This prostate-specific *PTEN* knockout (Cre-*PTEN*^−/−^-Luc, CPPL) mouse model was kindly provided by Dr. Pradip Roy-Burman and described previously [32]. CPPL mice were randomized into two groups (n = 4) and administered intraperitoneally with either 20 mg/kg sEphB4-alb or Phosphate-buffered saline (PBS) twice a week. Prostate tumors were monitored by bioluminescence imaging (Xenogen, Alameda, CA) before treatment and every 4 weeks after treatment. No blinding experiment for the investigators.

### Bioluminescence imaging (BLI)

Mice were given a single intraperitoneal (IP) injection of ketamine (50 mg/kg) and xylazine (10 mg/kg) followed by intravenous (IV) injection of luciferin (50 mg/kg). After allowing 4.5 min for the proper distribution of luciferin, mice were placed in the chamber of an IVIS 200 optical imaging system (Xenogen, Alameda, CA). Photons were collected for a period of 1 min and images were analyzed using LIVING IMAGE software v. 2.50 (Xenogen, Alameda, CA). Signal intensity was quantified for defined regions of interest as photon count rate per unit body area per unit solid angle subtended by the detector (units of photons/s/cm^2^/steradian).

### Generation and genotyping of the conditional *EPHB4* knockout mice

*EPHB4* gene targeting vector was constructed to replace exons 2 to 3 and parts of intron 1-4 with a pEZ FRT Lox cassette (a gift from Dr. Robert Maxson) in murine embryonic stem (ES) cells. LoxP sites were inserted into intron 1-2 and intron 3-4, resulting in a deletion of 33167 bps, from HindIII to AccI including part of intron1-2 (2188 bps), exon 2 (71 bps), intron 2-3 (120 bps), exon3 (288 bps), and part of intron 3-4 (598 bps). The *lacZ* gene was fused in-frame with the *EPHB4* coding sequence at the start of exon 2. Two correctly targeted ES cell lines were identified by PCR and Southern blot and used to generate chimeric mice. After germline transmission, heterozygote mice were crossed with FLP transgenic mice to remove Neo. Then, Neo-less heterozygote mice were crossed with wild-type C57BL/6 background mice to remove FLP.

Mice homozygous for floxed *EPHB4* exon 2-3, *EPHB4*^*floxP/floxP*^ were crossed with the CMV-Cre strain obtained from the Jackson Laboratory (Bar Harbor, ME) and PB-Cre-*PTEN*-Luc mice, in which the Cre transgene is controlled by a modified probasin promoter (ARR2PB) [33]. Littermate controls lacking the Cre transgene were used in all experiments. All procedures were approved by Institutional Animal Care and Use Committee and performed in accordance with the Animal Welfare Act regulations.

Mice were genotyped by PCR. Mouse tail-tips were isolated and incubated overnight at 55 °C in lysis buffer (Cat# 102-T, VIAGEN Biotech, LA, CA) with 0.5 µg/mL proteinase K (Cat# 03-H5-801-001, Roche Diagnostics, Indianapolis, IN). Tail-tip samples were then incubated at 85 °C for 45 min before use. The forward primer1 (5′-TTCTCGCCTGCGCTACCTGAATG-3′) and the reverse primer2 (5′- ACCAGGGCTCCATTTCTAGGTCG -3′) were used to distinguish the wild-type and target alleles by amplifying the flanking loxP sites. The forward primer (5′- GATCCTGGCAATTTCGGCTAT-3′) and the reverse primer (5′- TTGCCTGCATTACCGGTCGAT -3′) were used to detect the Cre transgene. Genomic DNA fragments were amplified at 95 °C for 5 min, then 95 °C for 45 sec, 58 °C for 40 sec, and 72 °C for 60 sec for 36 cycles, then 72 °C for 5 min. For detection of the exon 5 deletion, genomic DNA samples were isolated from different mouse organs using similar methods as for mouse tail-tips. The forward primer (5′- TAGGCTGGGCAGTGCTGTTCTGG -3′), and reverse primer, (5- CTCCTGTAGTCCAAGCTGGTCTC -3′) were used to detect exon 2-3 deletion.

### Antibodies and other reagents

Antibodies against P110α, P110β, P110γ, P110δ, p85 (PI3K subunits, rabbit monoclonal), Akt, phosphorylated Akt (Thr308; Ser473), S6, phosphorylated S6 (Ser240/244), ERK1/2 (Thr202/Tyr204), phosphorylated p38, p38, androgen receptor, PTEN, EphB4, C-Myc were from Cell Signaling (Danvers, MA). β-actin was from Sigma (St Louis, MO) and GAPDH (mouse monoclonal) antibody was from Millipore (Temecula, CA). Anti- Androgen receptor, Anti-Ki67 and anti-ephrin-B2 antibodies were from Abcam (Cambridge, MA) and anti-PtdIns(3,4,5)P3 antibody was from Echelon Biosciences (Salt Lake City, UT). Antibodies to other Eph receptors and ligands were obtained from R&D Systems (Minneapolis, MN). Horseradish peroxidase (HRP) and IRDye conjugated secondary antibodies were from Rockland (Gilbertsville, PA). c-Myc inhibitor 10058-F4 was from Thermo Fisher (Waltham, MA).

Complementary DNA (cDNA) encoding mouse EphB4 representing the entire extracellular domain was cloned upstream of the mature mouse serum albumin pCR-script and placed into the mammalian expression vector under control of the cytomegalovirus (CMV) promoter stably expressed in the Chinese hamster ovary (CHO) cell line. The expressed sEphB4-alb fusion protein was purified to homogeneity as described previously [30, 31].

Prostate adenocarcinoma tissue microarray, containing 80 cases of adenocarcinoma, 8 adjacent normal prostate tissue, and 8 normal prostate tissue (192 duplicate cores) was obtained from Biomax Cat# PR1921b (Derwood, MD).

### Western blotting

For Western blot, 20 µg of whole-cell lysates were run on 4-20% Tris-glycine gradient gel (Bio-Rad, Hercules, CA) and transferred onto nitrocellulose membrane (Bio-Rad, Hercules, CA). The membrane was blocked with 5% non-fat dry milk in TBS and 0.05% Tween-20 (TBST) for 40 min, and then incubated with 1 µg/ml primary antibody at 4 °C overnight. The membrane was washed three times for 10 min each and incubated with secondary HRP-labeled or IRDye labeled secondary antibody for 40 min. After washing three times with TBST, HRP signal was detected using SuperSignal West Femto Maximum Sensitivity chemiluminescent substrate (Thermo Scientific, Waltham, MA) and IRDye signal was detected by Odyssey (LICOR, Lincoln, NE).

### Immunofluorescence and immunohistochemistry

For immunofluorescence, fresh frozen tissue embedded in OCT was sectioned at 5 µm, fixed in phosphate-buffered 4% paraformaldehyde, and washed in PBS. Sections were then blocked with goat serum and incubated with primary antibody overnight at 4 °C. After washing in PBS, antibody binding was localized with AlexaFluor conjugated appropriate secondary antibodies (Invitrogen, Carlsbad, CA). Nuclei were counterstained with DAPI. Images were obtained with a Nikon Eclipse 80i fluorescence microscope and Meta Morph imaging series system. Tissues were also processed for apoptosis analysis with TdT-mediated dUTP nick-end labeling (TUNEL) assay kit (Promega, Madison, WI) following manufacturers’ instructions.

For immunohistochemistry, the frozen sections were fixed with 3% formaldehyde for 15 min at room temperature, following by two PBS washes. The sections were treated with 3% H_2_O_2_ for 10 min, blocked with goat serum for 1 h, and incubated with primary antibody overnight at 4 °C. The sections were then washed with PBS and processed with ABC kit (Vector labs, Burlingame, CA). The images were obtained with an Olympus BX51 microscope and Image-pro plus 6.0 system.

Four representative pictures were taken for each sample and quantification was performed with Image J (NIH). P value was determined by an unpaired 2-tail student T-test.

### Cell lines and culture

PC3, TRAMP-C2, HT29, Reh cell lines were obtained from the American Type Culture Collection. C4-2B cell was kindly provided by Michael Stallcup (University of Southern California), and 22Rv1, Raji and K562 cell lines were kindly provided by Dr. Akil Merchant (University of Southern California). All these cells were propagated in RPMI-1640 supplemented with 10% FBS, 100 units/mL of penicillin, and 100 μg/mL streptomycin from Cellgro (Thomas Scientific, Swedesboro, NJ). These cell lines have been validated by HLA typing and molecular phenotyping relative to the respective primary tumors.

### Murine tumor transplant models

The TRAMP-C2 cells were propagated, collected after trypsin digestion, and resuspended in serum-free medium. The 2 ×10^6^ TRAMP-C2 cells were injected subcutaneously in the flanks of male 8-week-old C57/BL6 mice. Tumor growth was measured 3 times a week, and volume was estimated as 0.52 x length x width^2^. Once tumors were established ( ~ 100mm^3^), animals were distributed into sEphB4-alb and control PBS groups (n = 8). Each group was treated by intraperitoneal injection of 20 mg/kg of sEphB4-alb 3 times a week. At the end of the experiment, mice were sacrificed for tissue analysis. All procedures were approved by University of Southern California institutional animal care and use committee and performed in accordance with the Animal Welfare Act regulations. No blinding experiment for the investigators.

### In situ hybridization

In situ hybridization was performed as described previously with modifications [34]. Specifically, sectional in situ was performed using ISH kit (Biochain, Hayward, CA) according to the manufacturer’s protocol. DIG-labeled antisense and sense probes were synthesized by in vitro transcription using T7 and T3 RNA polymerase (Promega, San Luis Obispo, CA). A 0.7 kb PCR fragment was amplified from the plasmid (BC076426) containing the full-length cDNA of mouse EphB4 and subcloned into pCR4.0 TOPO vector (Invitrogen, Carlsbad, CA). Images were obtained using an Olympus BX51 microscope equipped with a QImaging Retiga 2000R camera.

### Quantitative RT-PCR

Total RNA was extracted using RNeasy mini kit (Qiagen, Valencia, CA) from mouse prostate tissue. First-strand cDNA was synthesized from 2 µg of total RNA with the kit from Fermentas (Thermo Fisher, Waltham, MA) and then quantitative PCR was performed on the MX3000P real-time PCR system (Stratagene, La Jolla, CA) using Brilliant II SYBR Green QPCR Mastermix (Stratagene, La Jolla, CA) according to the manufacturer’s instructions. All reactions were performed in triplicate. The amplification signals were normalized to β-actin. For assessing EphB4 siRNA knockdown effect, total mRNA was extracted from cultured cells transfected with EphB4 siRNA or 3-base mis-match control siRNA (Control siRNA). Primer sequences of studied genes are shown in the Supplementary Table 1.

### siRNA and transfection

*EPHB4* siRNA (sequence was 5’-CCGGGAAGGUGAAUGUCAA-3’) was synthesized from Qiagen (Valencia, CA). Lipofectamine 2000 (Invitrogen, Carlsbad, CA) was used for siRNA transfection following manufacturer’s instructions.

### AKT constructs

The constructs wild-type Akt and myrAkt Δ4-129 (constitutively activated AKT), which contains a src myristylation signal sequence, were described previously [35]. These constructs were cloned into the pCMV-SPORT6 vector.

### In vitro AKT and phosphorylated AKT rescue experiment

C4-2B cells were seeded in 24-well plates at a density of 2×10^4^ cells/well in a total volume of 500 µL and, 24 h later, cells were transfected with *EPHB4* siRNA or control siRNA. Another 12 h later, these cells were further transfected with either AKT/pCMV-SPORT6 full length (BC020530.1, Open Biosystems), constitutional activated AKT/pCMV-SPORT6 or pCMV-SPORT6 plasmid. 2 days after treatment, cell viability was assessed using 3-(4,5-dimethylthiazol-2-yl)-2,5-diphenyltetrazolium bromide (MTT) as described previously [36]. Protein expression was confirmed by immunoblotting.

### Statistical analysis

The statistical significance between different samples or groups was determined using an unpaired two-tailed Student t test and chi-square. Results were considered significantly different if the P value was less than 0.05.

Vertebrate murine studies were conducted under the animal welfare committee approved protocol- IACUC- 20421.

Human tumor tissue arrays were purchased from a third-party vendor. No human research participant images are included in the manuscript.

All methods were performed in accordance with the relevant guidelines and regulations.

## Results

### EphB4 and ephrin-B2 are induced in prostate cancer

**Fig. 1:**
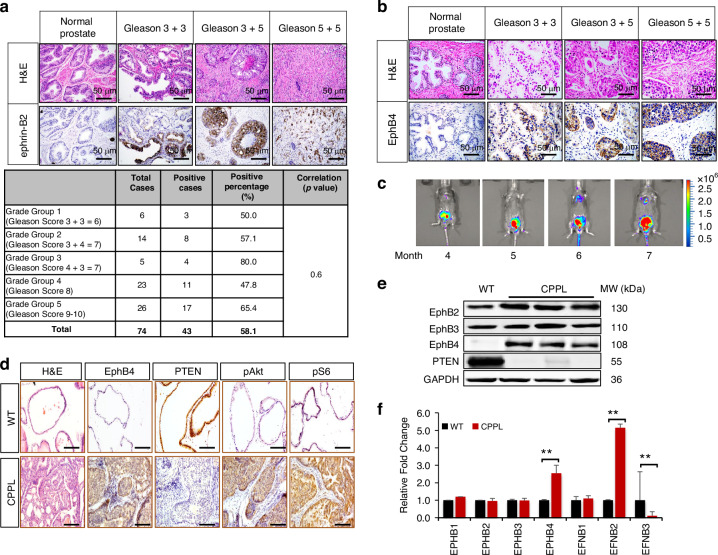
Induction of EphB4-ephrin-B2 in prostate cancers. **a** Hematoxylin and eosin stain (H&E), and immunohistochemistry (IHC) stain of ephrin-B2 and (**b**) EphB4 in one prostate cancer tissue microarray, containing 80 cases of adenocarcinoma, 8 each of adjacent normal prostate tissue and normal prostate tissue, duplicate cores per case (Biomax Inc. PR1921b). Scale bar = 50 µm. Top bar indicates the Gleason scores of prostate cancer. Table indicates subgroup analysis of ephrin-B2 expression level based on different Gleason scores. 6 prostate cancer cases was not able to be scored. There is no significant difference between Gleason scores, *p* = 0.6 (chi-square test). **c** Growth of primary prostate cancers in PTEN-null mice was followed noninvasively by bioluminescence imaging (BLI). Colors indicate the intensity of luciferase signal. Blue denotes weak signal; Green and yellow denote intermediate signal; Red denotes strong signal. **d** H&E and IHC of EphB4, PTEN, phosphorylated AKT (pAKT), phosphorylated S6 (pS6) protein level in wild-type (WT) mice (6-month age) prostate glands *versus* Cre-*PTEN*^−/−^-Luciferase (CPPL) mice prostate glands. All lobes of the prostate gland, uniformly expressed. The anterior prostate lobe was represented. Scale bar = 50 µm. **e** Western blot of EphB4, EphB2, EphB3 and PTEN protein level in one wild-type (WT) and three CPPL mice. Glyceraldehyde 3-phosphate dehydrogenase (GAPDH) was probed to ensure equal loading. MW (kDa): molecular weight (kilodalton). **f** Quantitative RT-PCR of EPHB4, EPHB1, EPHB2, EPHB3, EFNB1, EFNB2, and EFNB3 RNA levels were measured in wild-type (blue) and CPPL mice (red). Results are shown as gene expression relative fold change compared to wild-type (WT). Error bars represent standard deviation (s.d.). These experiments were repeated at least twice and similar results were obtained. P-value was calculated using two-tailed, unpaired Student’s t-test. **P < 0.002.

*EphB*-*EFNB* gene expression analysis was performed on 492 PC patient tissues using The Cancer Genome Atlas (TCGA) database. Among the EphB receptor subfamily, EphB4 and its cognate ligand ephrin-B2 had the highest levels (Supplementary Fig. 1). EphB4 protein levels have previously been reported in prostate cancer tissue [20, 21]. We thus analyzed ephrin-B2 expression in human PC. Among surgically resected 74 prostate cancers and 16 normal prostate tissues, ephrin-B2 was induced in 58% (43 out of 74) of the prostate tumors while normal prostate glands showed no expression (Fig. 1a, Supplementary Fig. 1A). ephrin-B2 expression did not correlate with Gleason grade (Fig. 1a), or tumor stage (Supplementary Table 2) in this small study of resectable prostate cancers, while ephrin-B2 induction begins early in the disease. EphB4 was similarly induced in these resectable tumors, while regulation in disease evolution needs investigation (Fig. 1b).

### EphB4 and ephrin-B2 are induced in a *PTEN*-null mouse model of prostate cancer

To determine if EphB4 expression is regulated by *PTEN* loss-of-function and activation of the PI3K pathway, we used a conditional knock out of *PTEN* in prostate epithelium using androgen responsive probasin-Cre, *PTEN*^f/f^ and monitored tumor real time using probasin driven luciferase (*cPTEN*^−/−^*L*) reporter [32, 37]. PC development begins at 9 weeks of age coinciding with adolescent increase in testosterone levels. Development and progression of prostate cancer was monitored noninvasively using bioluminescence imaging (BLI) [37, 38]. Beginning at 6 to 8 weeks of age, a cohort of 45 Cre-Probasin-*PTEN*^−/−^-Luc (CPPL) mice was monitored using BLI at intervals of 2 weeks for up to 52 weeks. Luciferase expression representing prostate cancer burden increased over time (Fig. 1c). EphB4 expression was measured in mouse prostate gland tissue by immunohistochemistry (IHC), and western blotting. An increase in EphB4 expression was seen in the tumor, while no expression was seen in normal prostate gland (Fig. 1d and e). We also examined the expression of other EphB receptor family members (EphB1, EphB2, EphB3) with no change in normal and prostate cancer (Fig. 1e). Quantitative RNA measurement showed increase in EphB4, without appreciable change in EphB1, EphB2 and EphB3 (Figs. 1e and 1f). Among the EphB ligands, EFNB2 had the highest expression (Supplementary Fig. 1B). Thus, *PTEN* loss and thus induction of PI3K pathway results in elevated EphB4-ephrin-B2 receptor-ligand axis.

### EphB4 is required for tumor initiation in a *PTEN*-null mouse model of prostate cancer

**Fig. 2:**
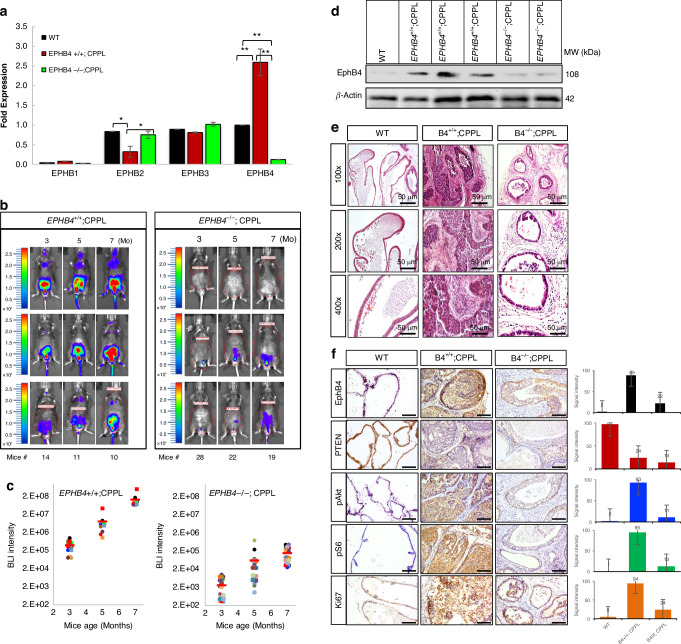
EphB4 is required for *PTEN* knockout prostate cancer initiation. **a** Quantitative RT-PCR of *EPHB4, EPHB1, EPHB2*, and *EPHB3* RNA levels were measured in wild-type mice (both *EPHB4* and *PTEN* are wild-type, blue), CPPL mice (CPPL mice with wild-type *EPHB4*, red), and *EPHB4* homo-CPPL mice (CPPL mice with homozygous deletion of *EPHB4*, purple) prostate tissues. Gene expression was normalized against β-actin. Error bars represent standard deviation (s.d.). These experiments were repeated at least three times and similar results were obtained. P-value was calculated using two-tailed, unpaired Student’s t-test. *P < 0.05; **P < 0.002. **b** BLI of luciferase expression in live mice imaging over 3, 5, and 7 months of age. Left panel shows 3 out of 14 CPPL mice with wild-type *EPHB4* (*EPHB4*^+/+^;CPPL). Right panel shows 3 out of 29 CPPL mice with homozygous *EPHB4* (*EPHB4*^–/–^;CPPL). Color bars indicate intensity of luciferase signal. Blue denotes weak signal; Green and yellow denote intermediate signal; Red denotes strong signal. **c** Quantitative measurement of total BLI signal intensity of 14 *EPHB4*^+/+^; CPPL mice and 29 *EPHB4*^–/–^;CPPL mice. Blue: 3 months old; red: 5 months; green: 7 months. BLI intensity is labeled on the top of each bar. Error bars represent s.d. **d** Western blot of EphB4 was performed on tumors harvested from the prostate gland of mice. β-actin was probed to ensure equal loading. These experiments were repeated at least three times and similar results were obtained. **e** H&E stain of prostate glands from wild-type, *EPHB4*^+/+^;CPPL and *EPHB4*^-/-^;CPPL mice under magnifications of 100×, 200× and 400×, respectively. **f** IHC stain of EphB4, PTEN, pAKT, pS6 and Ki67 in prostate tissues. Scale bar = 50 µm. Signal intensities shown on right panel were quantified based on positive staining pixel dots using ImageJ (NIH) software. All error bars represent s.d. WT: wild-type both *EPHB4* and *PTEN*; *EPHB4*^+/+^;CPPL: *EPHB4* wild-type *PTEN*-null mice; *EPHB4*^–/–^;CPPL: *EPHB4*-homozygote *PTEN*-null mice. All experiments were repeated at least three times and similar results were obtained.

**Fig. 3:**
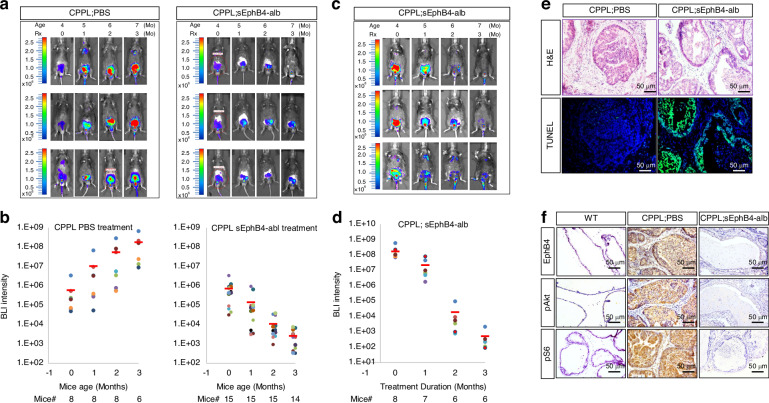
EphB4 is required for *PTEN* knockout prostate cancer progression. **a** BLI of luciferase expression in live mice at age 4, 5, 6, and 7 months corresponding to treatment length of 0, 1, 2, and 3 months, respectively. Left panel: CPPL treated with PBS (control). Right panel: CPPL treated with soluble EphB4-albumin (sEphB4-alb). Three mice from each group are shown. Color bars indicate the intensity of luciferase signal. **b** Quantitative measurement of total BLI signal intensity of 8 CPPL;PBS-treated mice and 15 CPPL;sEphB4-alb treated mice. BLI intensity is shown on the top of each bar. **c** BLI of live mice at age 7, 8, 9, and 10 months corresponding to treatment with sEphB4-alb at 0, 1, 2, and 3 months, respectively. Three mice are shown. Color bars indicate the intensity of luciferase signal. **d** Quantitative measurement of total BLI signal intensity of 8 CPPL;sEphB4-alb treated mice. Blue: 0-month treatment. Red: 1-month treatment. Green: 2-month treatment. Purple: 3-month treatment. BLI intensity is shown on the top of each bar. Error bars represent s.d. **e** TUNEL immunoassay (lower panel) of prostate tissues from PBS treated vs. sEphB4-alb treated CPPL mice and H&E stain (upper panel) of the serial slides from the same tissue. **f** IHC stain of EphB4, pAKT and pS6 in wild-type (WT), PBS treated CPPL (CPPL;PBS) and sEphB4-alb treated CPPL (CPPL;sEphB4-alb) prostate tissues. All experiments were repeated at least three times and similar results were obtained. Scale bar = 50 µm.

We hypothesized that EphB4 is required for disease initiation. This was achieved by deleting *EPHB4* in prostate epithelium in the context of *PTEN* knock out prostate cancer mouse model. We thus generated a floxed allele of *EPHB4* spanning exon 2 and 3, for conditional *EPHB4* knockout (*EPHB4* CKO) in prostate epithelium similar to the *PTEN* deletion. *EPHB4* RNA levels were significantly reduced in homozygote knockout CPPL;*EPHB4*^*−/−*^ mice (Fig. 2a). Strikingly *EPHB4* deletion prevented the development of PC in more than half (16 of 29) of the *EPHB4* and *PTEN* knockout mice (*EPHB4*^*−/−*^;*PTEN*^*−/−*^), while much smaller tumors were seen in the remaining 13 mice. (Fig. 2b, Supplementary Fig. 2 and Supplementary Fig. 3a–c). As expected all *EPHB4* wild type CPPL mice had progressive increase in tumor burden (*CPPL*;*EPHB4*^wt/wt^) (Fig. 2c). Tumors harvested from the *EPHB4* wild type and *PTEN*-null mice showed elevated levels of EphB4, but only a minimal signal was detected in prostate glands from wild-type *(CPPL;EPHB4*^wt/wt^) and *EPHB4*-knockout CPPL mice (*CPPL;EPHB4*^*−/−*^) (Fig. 2d). Dense tumors with loss of normal architecture were observed in the *PTEN* null mice (CPPL mice) (Fig. 2e). Compared to the wild-type control group, *EPHB4*-null group (*CPPL*; *EPHB4*^*−/−*^) mice had increased apoptosis and low proliferation in the prostate glands (Figs. 2f, 3e). *EPHB4* and *PTEN*-null mice showed marked reduction in phosphorylated AKT (pAKT), and phosphorylated S6 (pS6) (Fig. 2f).

### Soluble EphB4 decoy receptor precludes tumor development in *PTEN*-null prostate

We next utilized a pharmacological agent for orthogonal method to verify the significance of EphB4-ephrin-B2 pathway, using a murine version of sEphB4-alb fusion protein that binds ephrin-B2 and blocks cell-cell ephrin-B2-EphB4 binding leading to bidirectional signaling [31, 39]. sEphB4-alb also blocks the interaction of endogenous ephrin-B2 other lower affinity members of EphB family [28, 40]. To test tumor if EphB4-ephrin-B2 pathway is relevant for tumor initiation, CPPL mice were treated from age 8 to 28 week intra-peritoneally (IP) three times a week. Tumor development, measured by quantitative BLI, was markedly reduced in all 15 treated mice. On average, BLI in the treated group was nearly 70 thousand -fold lower than the control group after 3 months of therapy (*P* < 0.001) (Fig. 3a and b, Supplementary Fig. 4a–c). Tumor weight was similarly smaller in sEphB4-alb treated group than the control mice (Supplementary Fig. 4d).

We next studied the efficacy of sEphB4-alb in established tumors. Eight mice were treated per group with either sEphB4-alb or PBS for 12 weeks. Tumors regressed in all 8 mice in the drug treatment group, with complete regression in 5 mice (Fig. 3c and d Supplementary Fig. 5a, b). Imaging intensity progressively increased in the control group. BLI signal declined from 1.68E + 08 to 5.13E + 02 (decline >300 thousand times) in the treatment cohort. Prostate glands harvested at the end of the study showed increased apoptosis in sEphB4-alb treated CPPL mice compared to the control PBS treated group (Fig. 3e). Both pAKT and pS6K were also markedly lower in the sEphB4-alb treated group (Fig. 3f).

### sEphB4-alb efficacy in castration-resistant prostate cancer

**Fig. 4:**
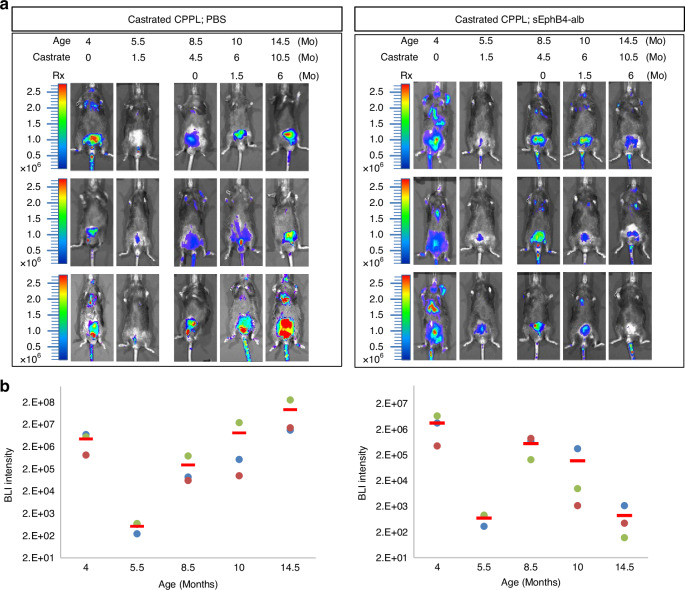
Castration resistant tumor sensitive to EphB4 blockage. **a** BLI of luciferase expression in live mice at 4 months, 5.5-months, 8.5-months, 10-months and 14.5-months of age corresponding to time post-castration of 0, 1.5, 4.5, 6, and 10.5 months, respectively. Mice were treated with either PBS or sEphb4-alb from 8.5 months of age. Left panel: CPPL treated with PBS. Right panel: CPPL treated with sEphB4-alb. Three mice from each group are shown. Color bars indicate the intensity of luciferase signal. **b** Quantitative measurement of total BLI signal intensity of 8 CPPL;sEphB4-alb treated mice. BLI intensity is shown on the top of each bar. Error bars represent s.d.

Tumors resistant to androgen deprivation poses a greater challenge with limited treatment options. We thus assessed the efficacy of sEphB4-alb in PTEN-null CPPL mice after castration. Mice were imaged to select those with tumor recurrence after initial regression. Mice were then treated with sEphB4-alb or control PBS. sEphB4-alb treatment led to tumor regression while control mice showed steady increase in the BLI (average 8.96E + 02 in sEphB4-alb group versus average 9.11E + 07 in control group after 24 weeks) (Fig. 4a and b, Supplementary Fig. 6). Thus, sEphB4-alb remains effective in castration resistant prostate cancer.

### sEphB4-alb efficacy in TRAMP-C2 xenograft model

TRAMP-C2 cells represent a prototypic murine androgen-dependent prostate cancer cell line derived from transgenic adenocarcinoma of the mouse prostate (TRAMP) model which express cytokeratin, E-cadherin, and androgen receptor but has wild type p53 [41]. We assessed the efficacy of sEphB4-alb in TRAMP-C2 xenograft C57BL/6 mice model. Tumor growth was significantly suppressed from week 20 to week 42 in sEphB4-alb treated group compared to control mice (Supplementary Fig. 7). Therefore, sEphB4-alb exhibits tumor inhibition in models other than monogenic PTEN deficiency.

### EphB4 regulates c-Myc in vivo

**Fig. 5:**
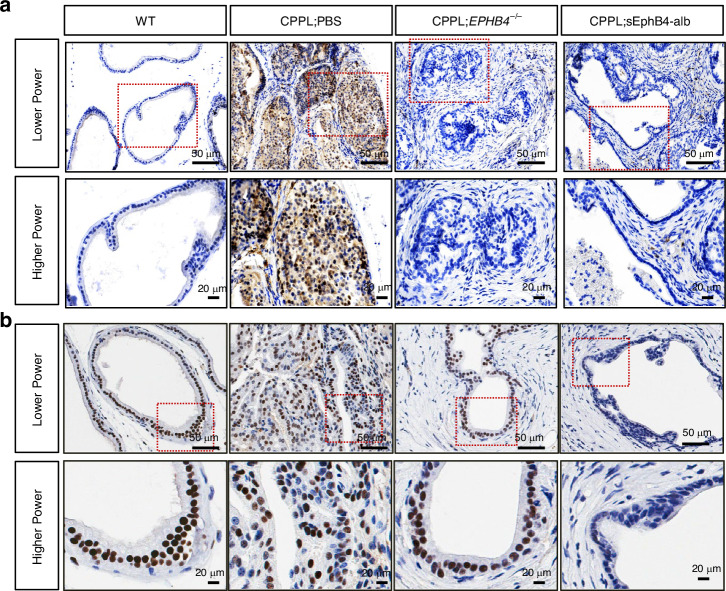
EphB4 regulates c-Myc and AR expression in vivo. IHC stain with (**a**) anti-Myc antibody and (**b**) anti-AR antibody (brown color) with in mouse prostate tissues from wild-type (WT), PBS-treated CPPL (CPPL;PBS), EphB4-knockout CPPL (CPPL;*EPHB4*^–/–^), and sEphB4-alb-treated CPPL (CPPL;sEphB4-alb) at lower power (400×, Scale bar = 50 µm), higher power (1000×, Scale bar = 20 µm) magnification. Dashed rectangle denotes the corresponding section shown at higher magnification.

Myc is often induced in prostate cancer and increases even further in castration resistant tumors [42]. Elevated c-Myc promotes tumor growth through global induction of transcription as well as regulation of androgen receptor. EphB4 has previously been shown to induce c-Myc [43]. In order to understand the sustained efficacy of sEphB4-alb even in castration resistant tumors, we tested the possibility that sEphB4-alb may reduce C-Myc level. C-Myc was markedly reduced in sEphB4-alb treated mice compared to high levels in control mice (Fig. 5a). Since c-Myc and PI3K pathway regulate AR, sEphB4-alb treated mouse tumors showed lower AR than controls (Fig. 5b). it is thus possible that sEphB4-alb retains efficacy in hormone response to CRPC even when c-Myc is upregulated.

### EphB4 regulates PI3K

**Fig. 6:**
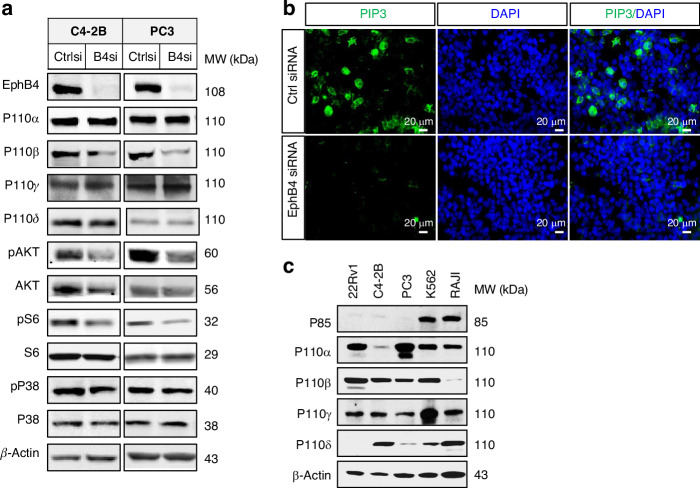
EphB4 regulates PI3K pathway in castration resistant prostate cancer cell lines. **a** Western blot of EphB4, P110α, P110β, P110γ, phosphorylated AKT (pAKT), AKT, phosphorylated S6 (PS6), S6, phosphorylated P38 (pP38), and P38 in C4-2B and PC3 human prostate cancer cell lines. β-actin was probed to ensure equal loading. Experiment was performed in triplicate. **b** Immunofluorescent (IF) stain of phosphatidylinositol (3,4,5) triphosphate (PIP3), DAPI (denotes nuclear staining) and merge of PIP3 and DAPI. Scale bar = 20 µm. **c** Western blot showed the expression level of P85, P110α, P110β, P110γ, and P110δ in different cancer cell lines 22Rv1, C4-2B, PC3, K562, and RAJI. Experiment was performed in triplicate.

Activation of the PI3K-AKT pathway through growth factor receptors, including epidermal growth factor (EGF)-EGFR, upregulates EphB4 expression [36, 44], especially with PTEN depletion (Fig. 1e). We examined if EphB4 had a feedback regulation of PI3K. EphB4 knockdown lowered EphB4 and the PI3K downstream markers pAKT (Thr308) and phosphorylated ribosomal protein S6 (pS6, Ser235/Ser236; Fig. 6a) in PC3 and castrate-resistant prostate cancer cell lines C4-2B and 22Rv1. EphB4 knockdown also reduced the activation of PI3K activity to convert phosphatidylinositol (3,4)-diphosphate (PIP2) to phosphatidylinositol (3,4,5) triphosphate (PIP3) (Fig. 6b), with no effect on the total or activated forms of MAPK and p38 (Fig. 6a). To test the specificity of EphB4 knockdown effect on PI3K activity, we performed rescue experiment using cell viability assay with ectopic expression of wild-type AKT and the constitutionally active form of AKT (∆-AKT) [35] (Supplementary Fig. 8a–c). Both wild-type AKT and ∆-AKT rescued PI3K inhibition from EphB4 siRNA (Supplementary Fig. 8b). These data suggest that EphB4 regulates the PI3K pathway at or above the PI3K level.

We next determined if EphB4 regulates other PI3K catalytic isoforms. Since 22Rv1 and C4-2B cells express PI3K p110α and β but low levels of p110γ and p110δ as positive controls (Fig. 6c). We thus tested specific inhibitors of each PI3K isoform for inhibition of the downstream signal, pS6. Inhibitors of PI3K p110α (BYL719) and p110β isoforms (GSK2636771) significantly reduced pS6 levels, while total S6 levels remain constant. In contrast, inhibitors of p110γ (IPI-549) or p110δ (GSK2269557) did not alter pS6 levels (Supplementary Fig. 9A), consistent with the tissue context and role of PI3K isoforms.

It has been suggested that p110β induces androgen receptor (AR) and AR downstream signaling and that specific inhibition of p110β may reduce AR levels [45–47]. We thus measured AR levels in PC cell lines treated with PI3K isoform inhibitors. AR levels declined only with inhibition of p110β, but not with inhibitors of p110α/γ/δ (Supplementary Fig. 9A). It is thus possible that EphB4 could regulate AR through the regulation of p110β. EphB4 knockdown indeed markedly reduced AR levels (Supplementary Fig. 10) and AR responsive genes, STAT-3, ARL4C and SGK1, in PC cell lines consistent with the in vivo effect of sEphB4-alb on AR (Supplementary Figs. 9B and 11A–B) [48]. These data suggest that EphB4-EphbrinB2 pathway may regulate AR through PI3Kβ.

### EphB4 regulates c-Myc in vitro

We wished to verify the in vivo data on c-Myc inhibition in vitro in cell lines representing gene alterations and resistance to hormone deprivation [49], since c-Myc is induced in PC as early as intra-epithelial neoplasia and overt tumor [42] but amplified in a majority of CRPC. C-Myc has profound effects in PC including the induction or ribosomal biosynthesis to induction of stem cell like signature. C-Myc induces PIM1, a serine/threonine kinase, and EZH2, a member of PRC2 polycomb repressor complex, by repressing mir26a to promote tumor proliferation and invasion [50]. EphB4 knockdown in PC cell line C4-2B showed reduction in c-Myc (Supplementary Fig. 11A-11B). These results were not surprising since EphB4 is known to induce c-Myc levels in colon cancer and loss of EphB4 reduces Myc levels [43]. Moreover, c-Myc plays a positive role in regulating androgen receptor and its splice variants in prostate cancer [51]. AR level was significantly reduced when C4-2B prostate cancer cells were treated with different concentrations of Myc inhibitor 10058-F4 (Supplementary Fig. 11C). These data suggest that EphB4 may also regulate AR through c-Myc.

## Discussion

*PTEN* loss of function mutations and alterations that upregulates PI3K are early events in PC [40, 52, 53]. *PTEN* conditional deletion in prostate epithelium induces PC in all mice and is used as a model system along with other genetic alterations to study tumor evolution. Previously, induction of EphB4 and role in tumor cell biology has been done in established tumors and tumor cell lines. Can EphB4 increase in the context of PTEN loss be related in part to the reduction in EphB4 degradation remains to be examined. Recent work shows that E3 ligase DTX3 variant DTX3c in complex with UBA1, UBE2N-and Cdc48/p97 binds EphB4 at its C terminus and induces degradation, in the context if IGFII/IR deprivation [54]. We have not tested if PTEN loss reduces IGF pathway or regulates components of DTX3c E3 complex. Increase in ephrinB2, the high affinity ligand for EphB4 increase in PTEN loss coincides with decrease in ephrinB3. It is unclear if there is regulatory pathway that promotes ephrinB2 and represses ephrinB3.

Current work first investigated the potential role of EphB4 in tumor initiation first using genetic model of *PTEN* and *EphB4* conditional deletion and subsequent studies of pharmacological inhibitor of the pathway in both tumor initiation and tumor progression. Conditional deleted *EphB4* in the prostate epithelium prevented tumor formation in over half the *PTEN* knock out mouse (Fig. 2b, c) and first demonstration that EphB4 is required for tumor initiation. Mice that did develop the tumor had a very slow progression (Fig. 2c), likely due to incomplete deletion of *EphB4*. PI3K signaling measured by the downstream activation of pAKT and pS6K also showed a marked decrease when EphB4 was deleted. Since EphB4 activation induces PI3K pathway [20, 40], it may enhance the amplitude of PI3K signaling downstream in *PTEN* deficient tumor cells. EphB4 is thus required for initiation of the tumor in the context of *PTEN* deficiency, either through cell autonomous signaling or through simultaneous induction of its high affinity cell membrane bound ephrin-B2. We thus tested sEphB4-alb, a pharmacological agent that blocks bidirectional signaling emanating from EphB4-ephrin-B2 interaction. sEphB4-alb blocked tumor initiation similar to the genetic model in experiments involving early pharmacological intervention in *PTEN* conditional deletion models at the age of 6-8 weeks. Male mice begin to produce androgen required to delete *PTEN*. We had expected that EphB4 is required to provide sustained tumor growth. This was confirmed by treatment intervention with sEphB4-alb when the tumor was already established. Rapid, deep and sustained regression of established tumors supports the sustained contribution of EphB4-ephrin-B2 in tumor progression (Fig. 3c, f). Efficacy of sEphB4-alb was also established in hormone refractory prostate cancer that recurred after castration when increase in c-Myc, amplification or mutation of androgen receptor, ligand independent constitutive activation of AR contribute to disease progression [55]. Thus EphB4-ephrin-B2 pathway has sustained role from initiation to progression in prostate cancer including androgen independence.

PI3K consists of four catalytic isoform (p110 α/β/γ/δ), each isoform hetero-dimerizes with regulatory subunit p85 to signal downstream and activate AKT and S6K. PI3K isoforms are expressed differentially in different tumor types, p110β being the major isoform induced in *PTEN* deficient or mutant PC [45, 46, 56]. We investigated if EphB4 has a feedback regulation of PI3K. first we studied which isoform induces EphB4 by using small molecule inhibitors of each isoform. p110β and to less extent PI3Kα reduced EphB4 and ectopic expression of p110β rescued the EphB4 levels. Conversely, inhibition of EphB4 reduced p110β levels. These findings indicate a context dependent regulation of PI3K-p110β and EphB4.

Role of EphB4 in regulating c-Myc has previously been described in colon cancer and likely to play role in other cancers with elevated EphB4 (Supplementary Fig. 12). c-Myc levels were markedly reduced in sEphB4-alb treated mice and loss of c-Myc was also confirmed in PC tumor cell lines following EphB4 knockdown. Since c-Myc and PI3K regulate AR levels, while AR regulation was not the focus of this study, it remains to be studied how EphB4 may regulate AR in prostate cancer.

In conclusion, EphB4-ephrin-B2 pathway is induced early in PC and appears to contribute to tumor initiation and progression. Mechanism of action studies revealed that EphB4-ephrin-B2 induces PI3K p110β, and PI3K p110β induces EphB4, indicative of a positive recruit. The EphB4-ephrin-B2 pathway is an important signaling mechanism that has been found to play a significant role in castration-resistant prostate cancer (CRPC). Responsiveness in CRPC shows downregulation of c-Myc and AR which are inter-dependent. Targeting EphB4-ephrin-B2 pathway in PC treatment is worthy of target positive clinical investigation. In addition, development of EphB4 or ephrinB2 specific antibodies conjugated with cytotoxic agents or T cell recruiters offer other opportunities for translation of these findings to the clinic.

## Supplementary information

EphB4-ephrin-B2 are targets in castration resistant prostate cancer

## Data Availability

We will review any request to make data available to investigators interested in the work included in the trial.
