# Supplementary material for: EphB4-ephrin-B2 are targets in castration resistant prostate cancer
Source: Br J Cancer. 2025 Mar 5;132(8):679–89. doi: 10.1038/s41416-025-02942-5 (PMC11997055; doi:10.1038/s41416-025-02942-5)
Supplement: Supplementary file 1 — EphB4-ephrin-B2 are targets in castration resistant prostate cancer [file 41416_2025_2942_MOESM1_ESM.pptx]

## Slide 1
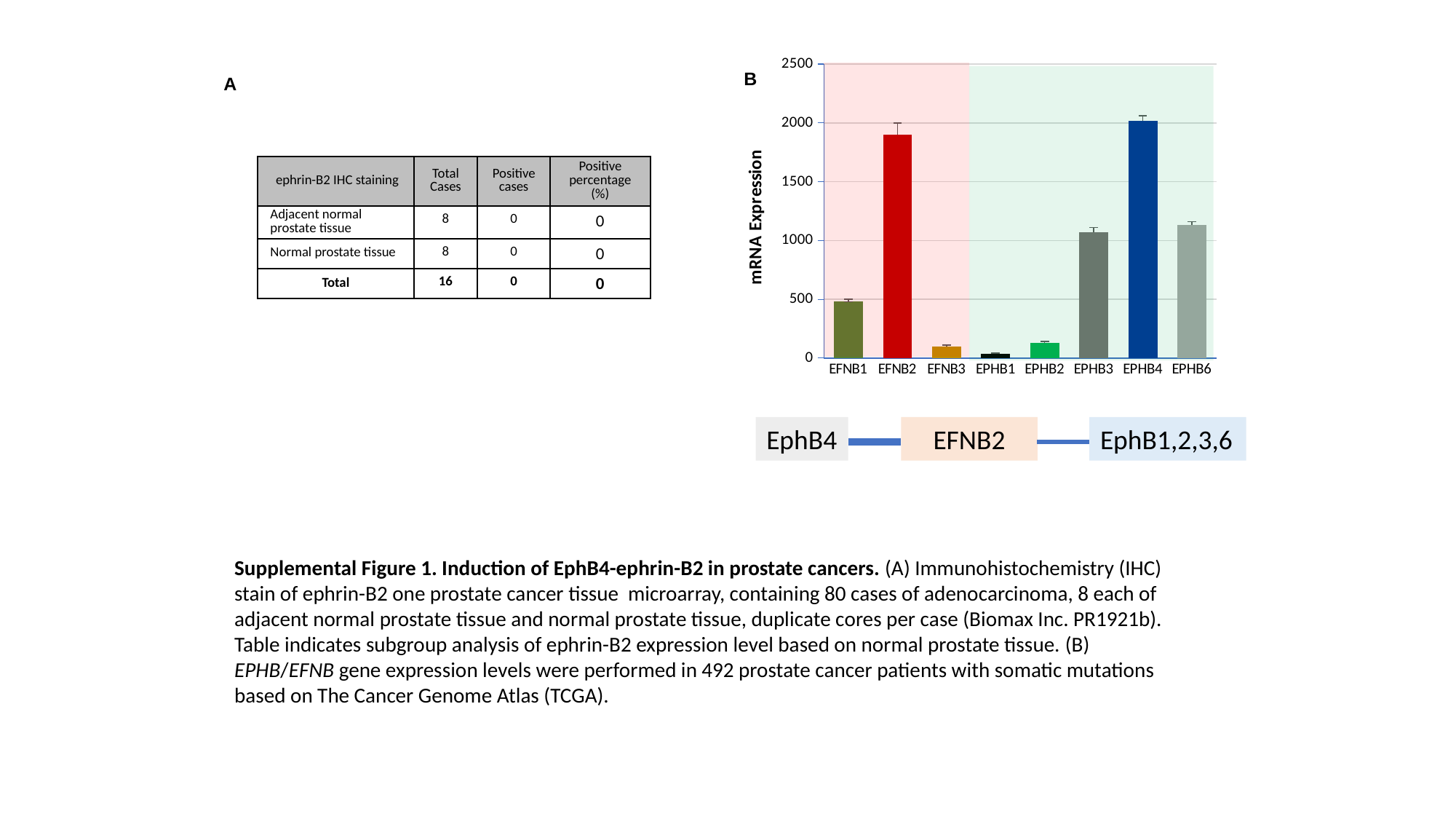

### Chart
| Category | Signal |
|---|---|
| EFNB1 | 480.0 |
| EFNB2 | 1900.0 |
| EFNB3 | 100.0 |
| EPHB1 | 35.0 |
| EPHB2 | 130.0 |
| EPHB3 | 1070.0 |
| EPHB4 | 2020.0 |
| EPHB6 | 1130.0 |B
A
| ephrin-B2 IHC staining | Total Cases | Positive cases | Positive percentage (%) |
| --- | --- | --- | --- |
| Adjacent normal prostate tissue | 8 | 0 | 0 |
| Normal prostate tissue | 8 | 0 | 0 |
| Total | 16 | 0 | 0 |
EphB4
EFNB2
EphB1,2,3,6
Supplemental Figure 1. Induction of EphB4-ephrin-B2 in prostate cancers. (A) Immunohistochemistry (IHC) stain of ephrin-B2 one prostate cancer tissue microarray, containing 80 cases of adenocarcinoma, 8 each of adjacent normal prostate tissue and normal prostate tissue, duplicate cores per case (Biomax Inc. PR1921b). Table indicates subgroup analysis of ephrin-B2 expression level based on normal prostate tissue. (B) EPHB/EFNB gene expression levels were performed in 492 prostate cancer patients with somatic mutations based on The Cancer Genome Atlas (TCGA).

## Slide 2
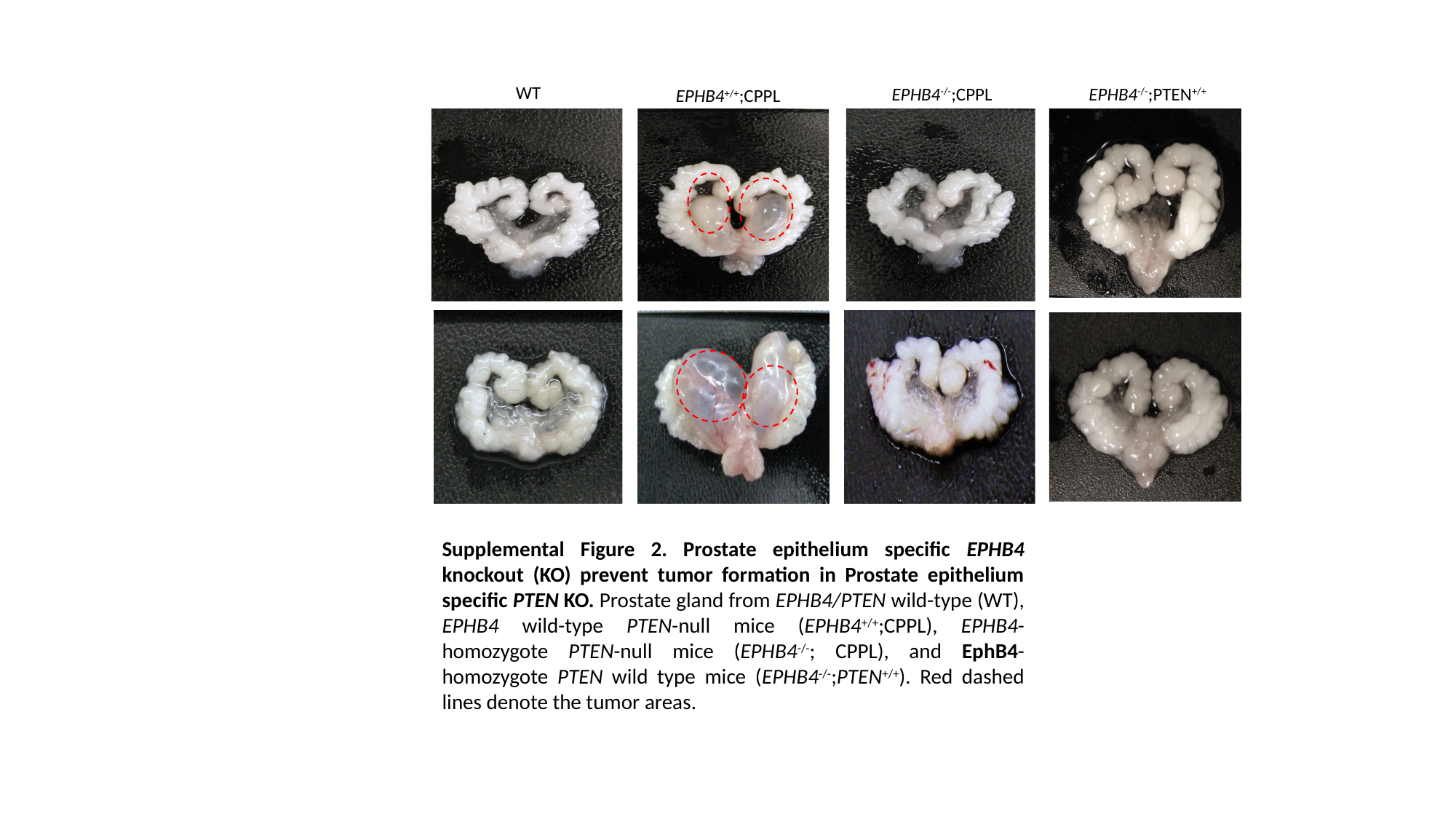

WT
EPHB4-/-;CPPL
EPHB4-/-;PTEN+/+
EPHB4+/+;CPPL
Supplemental Figure 2. Prostate epithelium specific EPHB4 knockout (KO) prevent tumor formation in Prostate epithelium specific PTEN KO. Prostate gland from EPHB4/PTEN wild-type (WT), EPHB4 wild-type PTEN-null mice (EPHB4+/+;CPPL), EPHB4-homozygote PTEN-null mice (EPHB4-/-; CPPL), and EphB4-homozygote PTEN wild type mice (EPHB4-/-;PTEN+/+). Red dashed lines denote the tumor areas.

## Slide 3
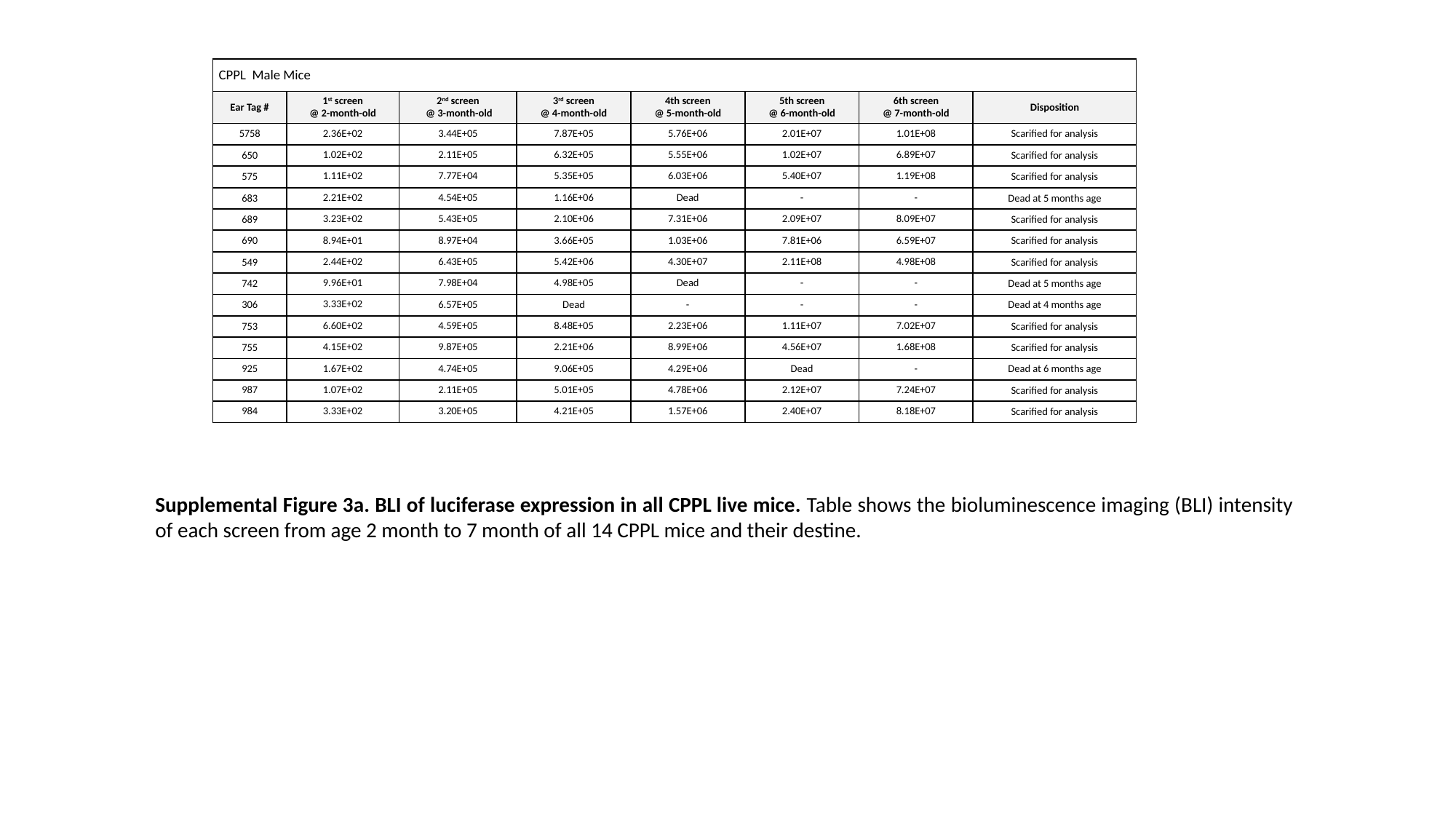

| CPPL Male Mice | | | | | | | |
| --- | --- | --- | --- | --- | --- | --- | --- |
| Ear Tag # | 1st screen @ 2-month-old | 2nd screen @ 3-month-old | 3rd screen @ 4-month-old | 4th screen @ 5-month-old | 5th screen @ 6-month-old | 6th screen @ 7-month-old | Disposition |
| 5758 | 2.36E+02 | 3.44E+05 | 7.87E+05 | 5.76E+06 | 2.01E+07 | 1.01E+08 | Scarified for analysis |
| 650 | 1.02E+02 | 2.11E+05 | 6.32E+05 | 5.55E+06 | 1.02E+07 | 6.89E+07 | Scarified for analysis |
| 575 | 1.11E+02 | 7.77E+04 | 5.35E+05 | 6.03E+06 | 5.40E+07 | 1.19E+08 | Scarified for analysis |
| 683 | 2.21E+02 | 4.54E+05 | 1.16E+06 | Dead | - | - | Dead at 5 months age |
| 689 | 3.23E+02 | 5.43E+05 | 2.10E+06 | 7.31E+06 | 2.09E+07 | 8.09E+07 | Scarified for analysis |
| 690 | 8.94E+01 | 8.97E+04 | 3.66E+05 | 1.03E+06 | 7.81E+06 | 6.59E+07 | Scarified for analysis |
| 549 | 2.44E+02 | 6.43E+05 | 5.42E+06 | 4.30E+07 | 2.11E+08 | 4.98E+08 | Scarified for analysis |
| 742 | 9.96E+01 | 7.98E+04 | 4.98E+05 | Dead | - | - | Dead at 5 months age |
| 306 | 3.33E+02 | 6.57E+05 | Dead | - | - | - | Dead at 4 months age |
| 753 | 6.60E+02 | 4.59E+05 | 8.48E+05 | 2.23E+06 | 1.11E+07 | 7.02E+07 | Scarified for analysis |
| 755 | 4.15E+02 | 9.87E+05 | 2.21E+06 | 8.99E+06 | 4.56E+07 | 1.68E+08 | Scarified for analysis |
| 925 | 1.67E+02 | 4.74E+05 | 9.06E+05 | 4.29E+06 | Dead | - | Dead at 6 months age |
| 987 | 1.07E+02 | 2.11E+05 | 5.01E+05 | 4.78E+06 | 2.12E+07 | 7.24E+07 | Scarified for analysis |
| 984 | 3.33E+02 | 3.20E+05 | 4.21E+05 | 1.57E+06 | 2.40E+07 | 8.18E+07 | Scarified for analysis |
Supplemental Figure 3a. BLI of luciferase expression in all CPPL live mice. Table shows the bioluminescence imaging (BLI) intensity of each screen from age 2 month to 7 month of all 14 CPPL mice and their destine.

## Slide 4
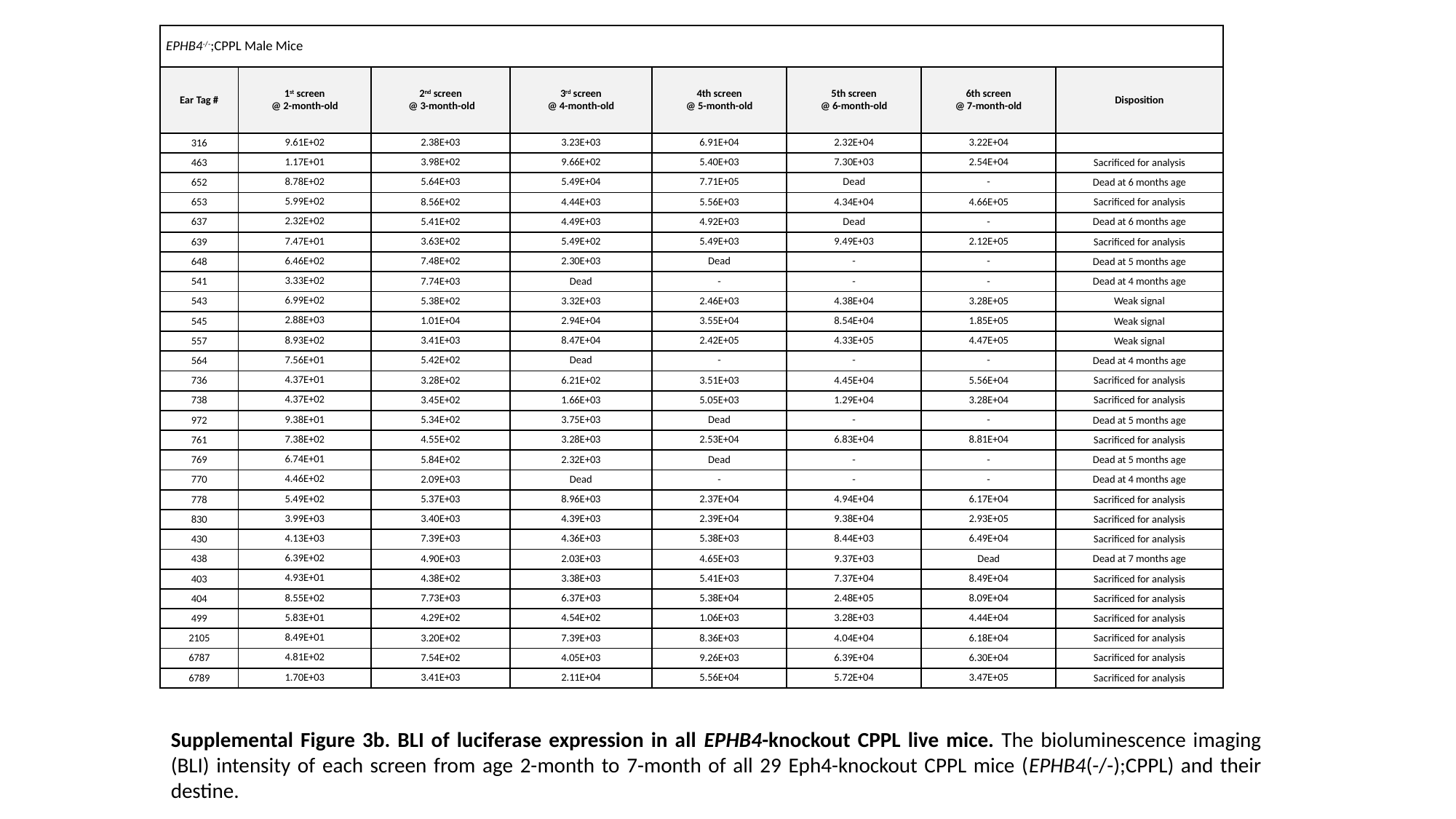

| EPHB4-/-;CPPL Male Mice | | | | | | | |
| --- | --- | --- | --- | --- | --- | --- | --- |
| Ear Tag # | 1st screen @ 2-month-old | 2nd screen @ 3-month-old | 3rd screen @ 4-month-old | 4th screen @ 5-month-old | 5th screen @ 6-month-old | 6th screen @ 7-month-old | Disposition |
| 316 | 9.61E+02 | 2.38E+03 | 3.23E+03 | 6.91E+04 | 2.32E+04 | 3.22E+04 | |
| 463 | 1.17E+01 | 3.98E+02 | 9.66E+02 | 5.40E+03 | 7.30E+03 | 2.54E+04 | Sacrificed for analysis |
| 652 | 8.78E+02 | 5.64E+03 | 5.49E+04 | 7.71E+05 | Dead | - | Dead at 6 months age |
| 653 | 5.99E+02 | 8.56E+02 | 4.44E+03 | 5.56E+03 | 4.34E+04 | 4.66E+05 | Sacrificed for analysis |
| 637 | 2.32E+02 | 5.41E+02 | 4.49E+03 | 4.92E+03 | Dead | - | Dead at 6 months age |
| 639 | 7.47E+01 | 3.63E+02 | 5.49E+02 | 5.49E+03 | 9.49E+03 | 2.12E+05 | Sacrificed for analysis |
| 648 | 6.46E+02 | 7.48E+02 | 2.30E+03 | Dead | - | - | Dead at 5 months age |
| 541 | 3.33E+02 | 7.74E+03 | Dead | - | - | - | Dead at 4 months age |
| 543 | 6.99E+02 | 5.38E+02 | 3.32E+03 | 2.46E+03 | 4.38E+04 | 3.28E+05 | Weak signal |
| 545 | 2.88E+03 | 1.01E+04 | 2.94E+04 | 3.55E+04 | 8.54E+04 | 1.85E+05 | Weak signal |
| 557 | 8.93E+02 | 3.41E+03 | 8.47E+04 | 2.42E+05 | 4.33E+05 | 4.47E+05 | Weak signal |
| 564 | 7.56E+01 | 5.42E+02 | Dead | - | - | - | Dead at 4 months age |
| 736 | 4.37E+01 | 3.28E+02 | 6.21E+02 | 3.51E+03 | 4.45E+04 | 5.56E+04 | Sacrificed for analysis |
| 738 | 4.37E+02 | 3.45E+02 | 1.66E+03 | 5.05E+03 | 1.29E+04 | 3.28E+04 | Sacrificed for analysis |
| 972 | 9.38E+01 | 5.34E+02 | 3.75E+03 | Dead | - | - | Dead at 5 months age |
| 761 | 7.38E+02 | 4.55E+02 | 3.28E+03 | 2.53E+04 | 6.83E+04 | 8.81E+04 | Sacrificed for analysis |
| 769 | 6.74E+01 | 5.84E+02 | 2.32E+03 | Dead | - | - | Dead at 5 months age |
| 770 | 4.46E+02 | 2.09E+03 | Dead | - | - | - | Dead at 4 months age |
| 778 | 5.49E+02 | 5.37E+03 | 8.96E+03 | 2.37E+04 | 4.94E+04 | 6.17E+04 | Sacrificed for analysis |
| 830 | 3.99E+03 | 3.40E+03 | 4.39E+03 | 2.39E+04 | 9.38E+04 | 2.93E+05 | Sacrificed for analysis |
| 430 | 4.13E+03 | 7.39E+03 | 4.36E+03 | 5.38E+03 | 8.44E+03 | 6.49E+04 | Sacrificed for analysis |
| 438 | 6.39E+02 | 4.90E+03 | 2.03E+03 | 4.65E+03 | 9.37E+03 | Dead | Dead at 7 months age |
| 403 | 4.93E+01 | 4.38E+02 | 3.38E+03 | 5.41E+03 | 7.37E+04 | 8.49E+04 | Sacrificed for analysis |
| 404 | 8.55E+02 | 7.73E+03 | 6.37E+03 | 5.38E+04 | 2.48E+05 | 8.09E+04 | Sacrificed for analysis |
| 499 | 5.83E+01 | 4.29E+02 | 4.54E+02 | 1.06E+03 | 3.28E+03 | 4.44E+04 | Sacrificed for analysis |
| 2105 | 8.49E+01 | 3.20E+02 | 7.39E+03 | 8.36E+03 | 4.04E+04 | 6.18E+04 | Sacrificed for analysis |
| 6787 | 4.81E+02 | 7.54E+02 | 4.05E+03 | 9.26E+03 | 6.39E+04 | 6.30E+04 | Sacrificed for analysis |
| 6789 | 1.70E+03 | 3.41E+03 | 2.11E+04 | 5.56E+04 | 5.72E+04 | 3.47E+05 | Sacrificed for analysis |
Supplemental Figure 3b. BLI of luciferase expression in all EPHB4-knockout CPPL live mice. The bioluminescence imaging (BLI) intensity of each screen from age 2-month to 7-month of all 29 Eph4-knockout CPPL mice (EPHB4(-/-);CPPL) and their destine.

## Slide 5
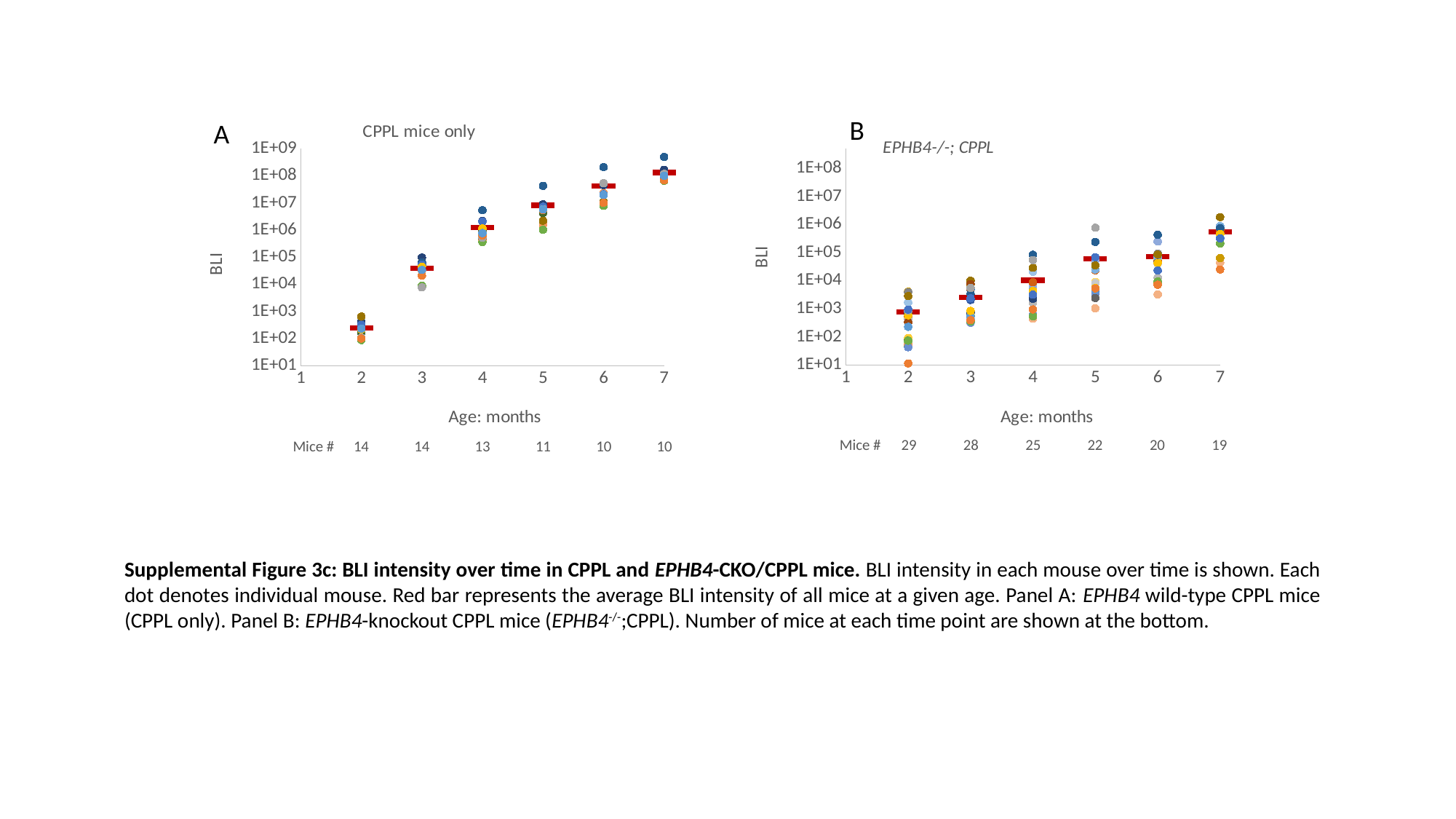

### Chart: CPPL mice only
| Category | 1 | 2 | 3 | 4 | 5 | 6 | 7 | 8 | 9 | 10 | 11 | 12 | 13 | 14 | AVG |
|---|---|---|---|---|---|---|---|---|---|---|---|---|---|---|---|
### Chart: EPHB4-/-; CPPL
| Category | 1 | 2 | 3 | 4 | 5 | 6 | 7 | 8 | 9 | 10 | 11 | 12 | 13 | 14 | 15 | 16 | 17 | 18 | 19 | 20 | 21 | 22 | 23 | 24 | 25 | 26 | 27 | 28 | 29 | AVE |
|---|---|---|---|---|---|---|---|---|---|---|---|---|---|---|---|---|---|---|---|---|---|---|---|---|---|---|---|---|---|---|B
A
| Mice # | 29 | 28 | 25 | 22 | 20 | 19 |
| --- | --- | --- | --- | --- | --- | --- |
| Mice # | 14 | 14 | 13 | 11 | 10 | 10 |
| --- | --- | --- | --- | --- | --- | --- |
Supplemental Figure 3c: BLI intensity over time in CPPL and EPHB4-CKO/CPPL mice. BLI intensity in each mouse over time is shown. Each dot denotes individual mouse. Red bar represents the average BLI intensity of all mice at a given age. Panel A: EPHB4 wild-type CPPL mice (CPPL only). Panel B: EPHB4-knockout CPPL mice (EPHB4-/-;CPPL). Number of mice at each time point are shown at the bottom.

## Slide 6
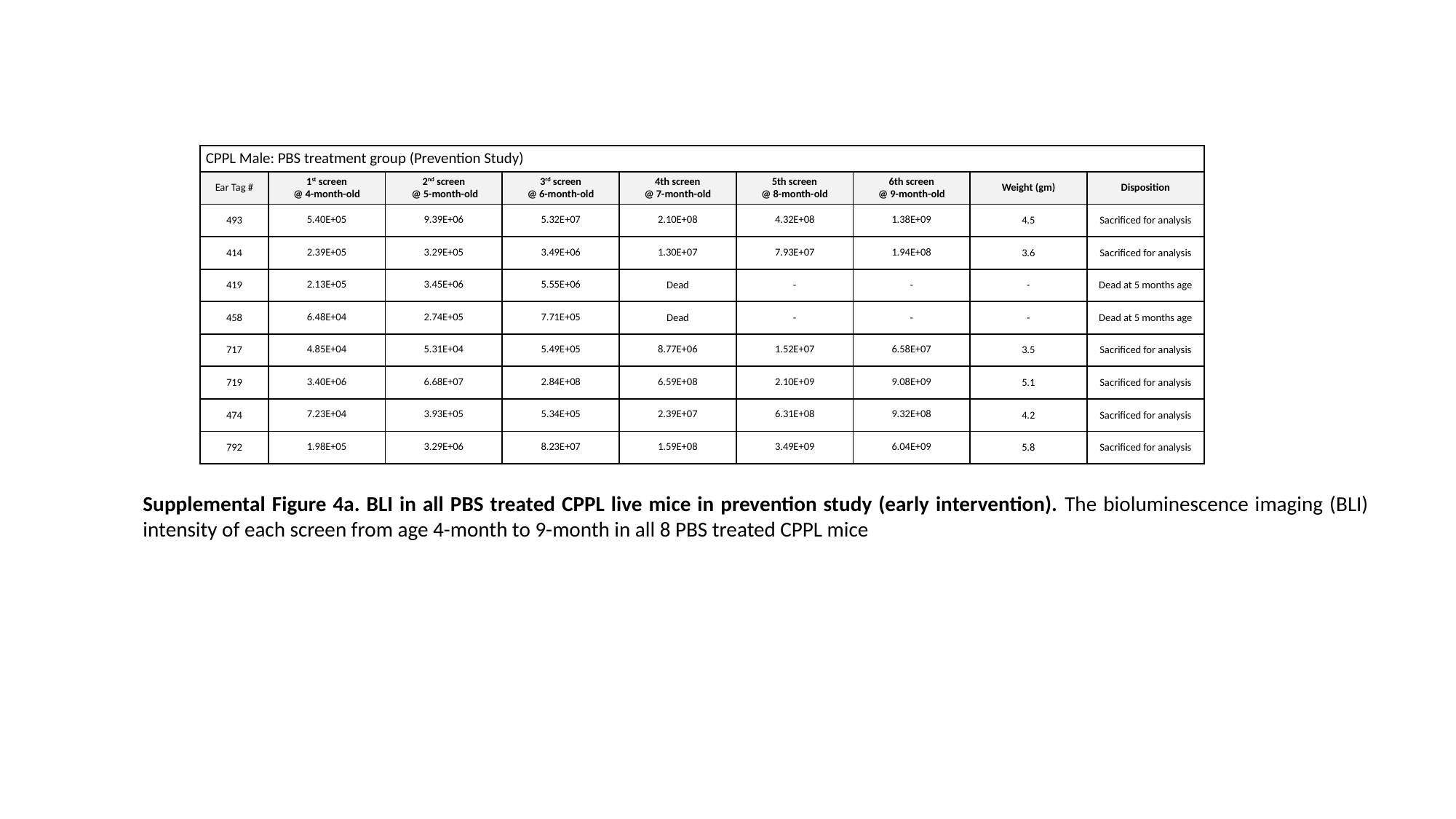

| CPPL Male: PBS treatment group (Prevention Study) | | | | | | | | |
| --- | --- | --- | --- | --- | --- | --- | --- | --- |
| Ear Tag # | 1st screen @ 4-month-old | 2nd screen @ 5-month-old | 3rd screen @ 6-month-old | 4th screen @ 7-month-old | 5th screen @ 8-month-old | 6th screen @ 9-month-old | Weight (gm) | Disposition |
| 493 | 5.40E+05 | 9.39E+06 | 5.32E+07 | 2.10E+08 | 4.32E+08 | 1.38E+09 | 4.5 | Sacrificed for analysis |
| 414 | 2.39E+05 | 3.29E+05 | 3.49E+06 | 1.30E+07 | 7.93E+07 | 1.94E+08 | 3.6 | Sacrificed for analysis |
| 419 | 2.13E+05 | 3.45E+06 | 5.55E+06 | Dead | - | - | - | Dead at 5 months age |
| 458 | 6.48E+04 | 2.74E+05 | 7.71E+05 | Dead | - | - | - | Dead at 5 months age |
| 717 | 4.85E+04 | 5.31E+04 | 5.49E+05 | 8.77E+06 | 1.52E+07 | 6.58E+07 | 3.5 | Sacrificed for analysis |
| 719 | 3.40E+06 | 6.68E+07 | 2.84E+08 | 6.59E+08 | 2.10E+09 | 9.08E+09 | 5.1 | Sacrificed for analysis |
| 474 | 7.23E+04 | 3.93E+05 | 5.34E+05 | 2.39E+07 | 6.31E+08 | 9.32E+08 | 4.2 | Sacrificed for analysis |
| 792 | 1.98E+05 | 3.29E+06 | 8.23E+07 | 1.59E+08 | 3.49E+09 | 6.04E+09 | 5.8 | Sacrificed for analysis |
Supplemental Figure 4a. BLI in all PBS treated CPPL live mice in prevention study (early intervention). The bioluminescence imaging (BLI) intensity of each screen from age 4-month to 9-month in all 8 PBS treated CPPL mice

## Slide 7
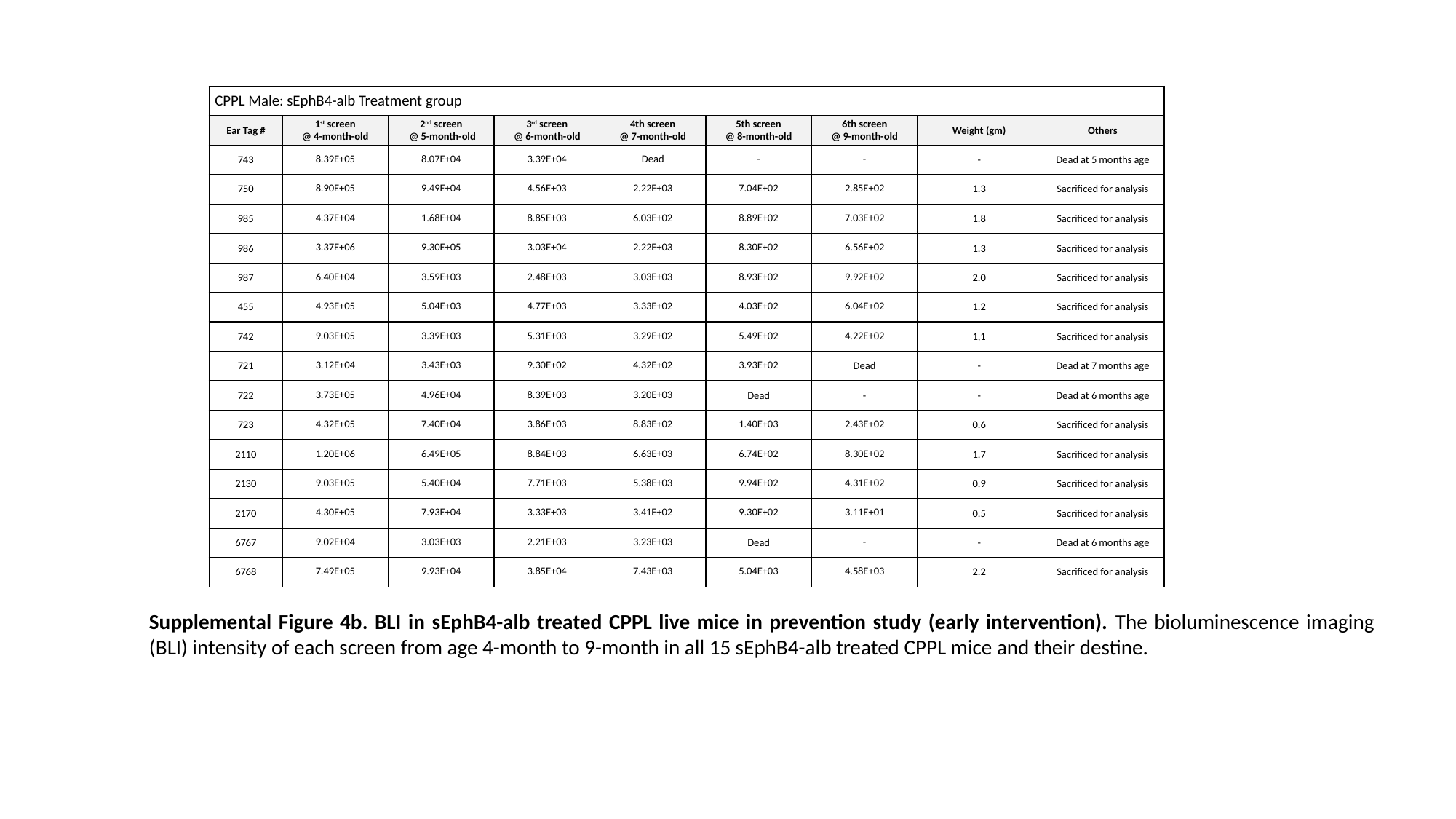

| CPPL Male: sEphB4-alb Treatment group | | | | | | | | |
| --- | --- | --- | --- | --- | --- | --- | --- | --- |
| Ear Tag # | 1st screen @ 4-month-old | 2nd screen @ 5-month-old | 3rd screen @ 6-month-old | 4th screen @ 7-month-old | 5th screen @ 8-month-old | 6th screen @ 9-month-old | Weight (gm) | Others |
| 743 | 8.39E+05 | 8.07E+04 | 3.39E+04 | Dead | - | - | - | Dead at 5 months age |
| 750 | 8.90E+05 | 9.49E+04 | 4.56E+03 | 2.22E+03 | 7.04E+02 | 2.85E+02 | 1.3 | Sacrificed for analysis |
| 985 | 4.37E+04 | 1.68E+04 | 8.85E+03 | 6.03E+02 | 8.89E+02 | 7.03E+02 | 1.8 | Sacrificed for analysis |
| 986 | 3.37E+06 | 9.30E+05 | 3.03E+04 | 2.22E+03 | 8.30E+02 | 6.56E+02 | 1.3 | Sacrificed for analysis |
| 987 | 6.40E+04 | 3.59E+03 | 2.48E+03 | 3.03E+03 | 8.93E+02 | 9.92E+02 | 2.0 | Sacrificed for analysis |
| 455 | 4.93E+05 | 5.04E+03 | 4.77E+03 | 3.33E+02 | 4.03E+02 | 6.04E+02 | 1.2 | Sacrificed for analysis |
| 742 | 9.03E+05 | 3.39E+03 | 5.31E+03 | 3.29E+02 | 5.49E+02 | 4.22E+02 | 1,1 | Sacrificed for analysis |
| 721 | 3.12E+04 | 3.43E+03 | 9.30E+02 | 4.32E+02 | 3.93E+02 | Dead | - | Dead at 7 months age |
| 722 | 3.73E+05 | 4.96E+04 | 8.39E+03 | 3.20E+03 | Dead | - | - | Dead at 6 months age |
| 723 | 4.32E+05 | 7.40E+04 | 3.86E+03 | 8.83E+02 | 1.40E+03 | 2.43E+02 | 0.6 | Sacrificed for analysis |
| 2110 | 1.20E+06 | 6.49E+05 | 8.84E+03 | 6.63E+03 | 6.74E+02 | 8.30E+02 | 1.7 | Sacrificed for analysis |
| 2130 | 9.03E+05 | 5.40E+04 | 7.71E+03 | 5.38E+03 | 9.94E+02 | 4.31E+02 | 0.9 | Sacrificed for analysis |
| 2170 | 4.30E+05 | 7.93E+04 | 3.33E+03 | 3.41E+02 | 9.30E+02 | 3.11E+01 | 0.5 | Sacrificed for analysis |
| 6767 | 9.02E+04 | 3.03E+03 | 2.21E+03 | 3.23E+03 | Dead | - | - | Dead at 6 months age |
| 6768 | 7.49E+05 | 9.93E+04 | 3.85E+04 | 7.43E+03 | 5.04E+03 | 4.58E+03 | 2.2 | Sacrificed for analysis |
Supplemental Figure 4b. BLI in sEphB4-alb treated CPPL live mice in prevention study (early intervention). The bioluminescence imaging (BLI) intensity of each screen from age 4-month to 9-month in all 15 sEphB4-alb treated CPPL mice and their destine.

## Slide 8
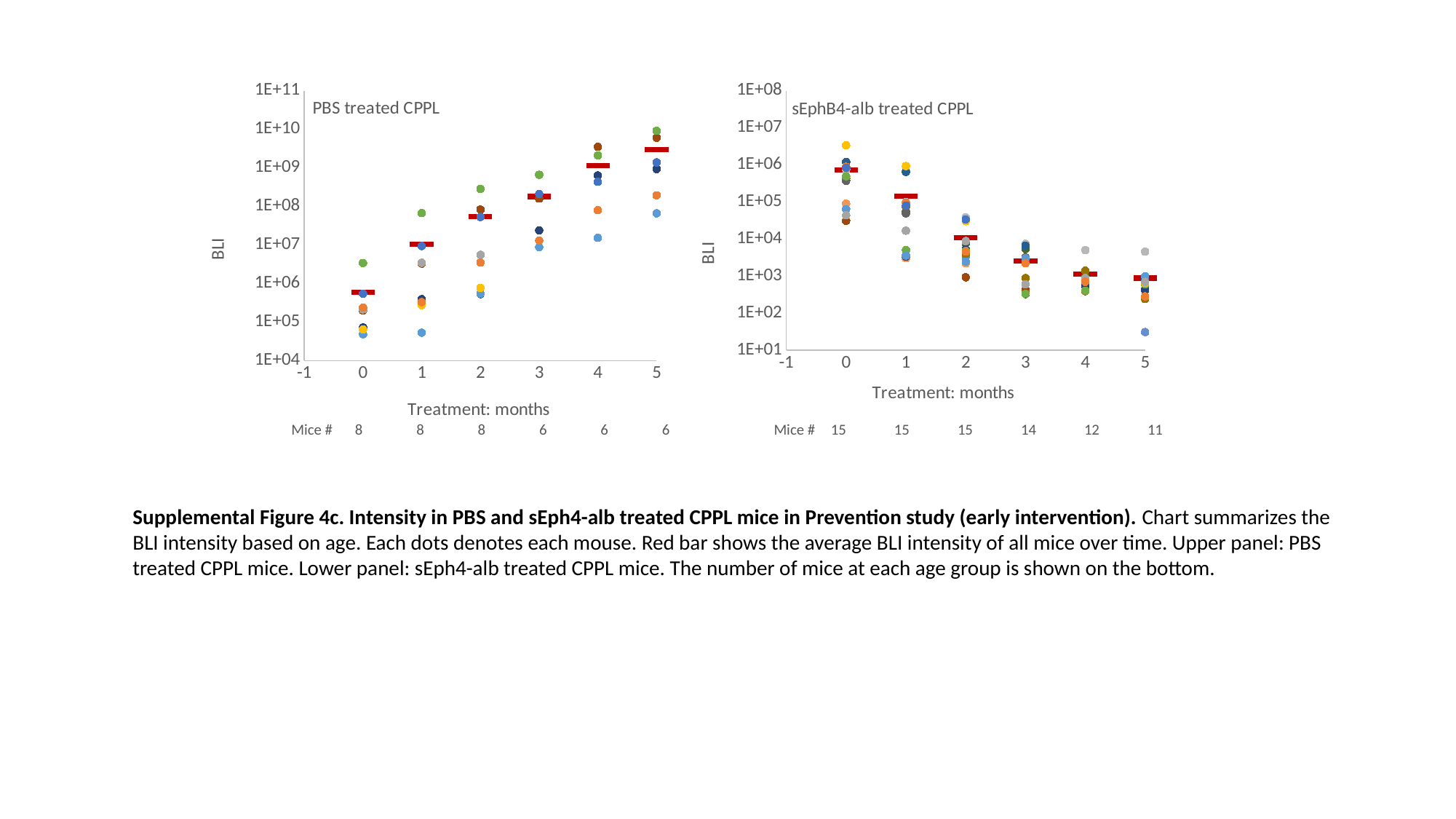

### Chart: PBS treated CPPL
| Category | 1 | 2 | 3 | 4 | 5 | 6 | 7 | 8 | AVE |
|---|---|---|---|---|---|---|---|---|---|
### Chart: sEphB4-alb treated CPPL
| Category | 1 | 2 | 3 | 4 | 5 | 6 | 7 | 8 | 9 | 10 | 11 | 12 | 13 | 14 | 15 | AVE |
|---|---|---|---|---|---|---|---|---|---|---|---|---|---|---|---|---|| Mice # | 8 | 8 | 8 | 6 | 6 | 6 |
| --- | --- | --- | --- | --- | --- | --- |
| Mice # | 15 | 15 | 15 | 14 | 12 | 11 |
| --- | --- | --- | --- | --- | --- | --- |
Supplemental Figure 4c. Intensity in PBS and sEph4-alb treated CPPL mice in Prevention study (early intervention). Chart summarizes the BLI intensity based on age. Each dots denotes each mouse. Red bar shows the average BLI intensity of all mice over time. Upper panel: PBS treated CPPL mice. Lower panel: sEph4-alb treated CPPL mice. The number of mice at each age group is shown on the bottom.
| Mice # | 8 | 8 | 8 | 6 | 6 | 6 |
| --- | --- | --- | --- | --- | --- | --- |
| Mice # | 15 | 15 | 15 | 14 | 12 | 11 |
| --- | --- | --- | --- | --- | --- | --- |

## Slide 9
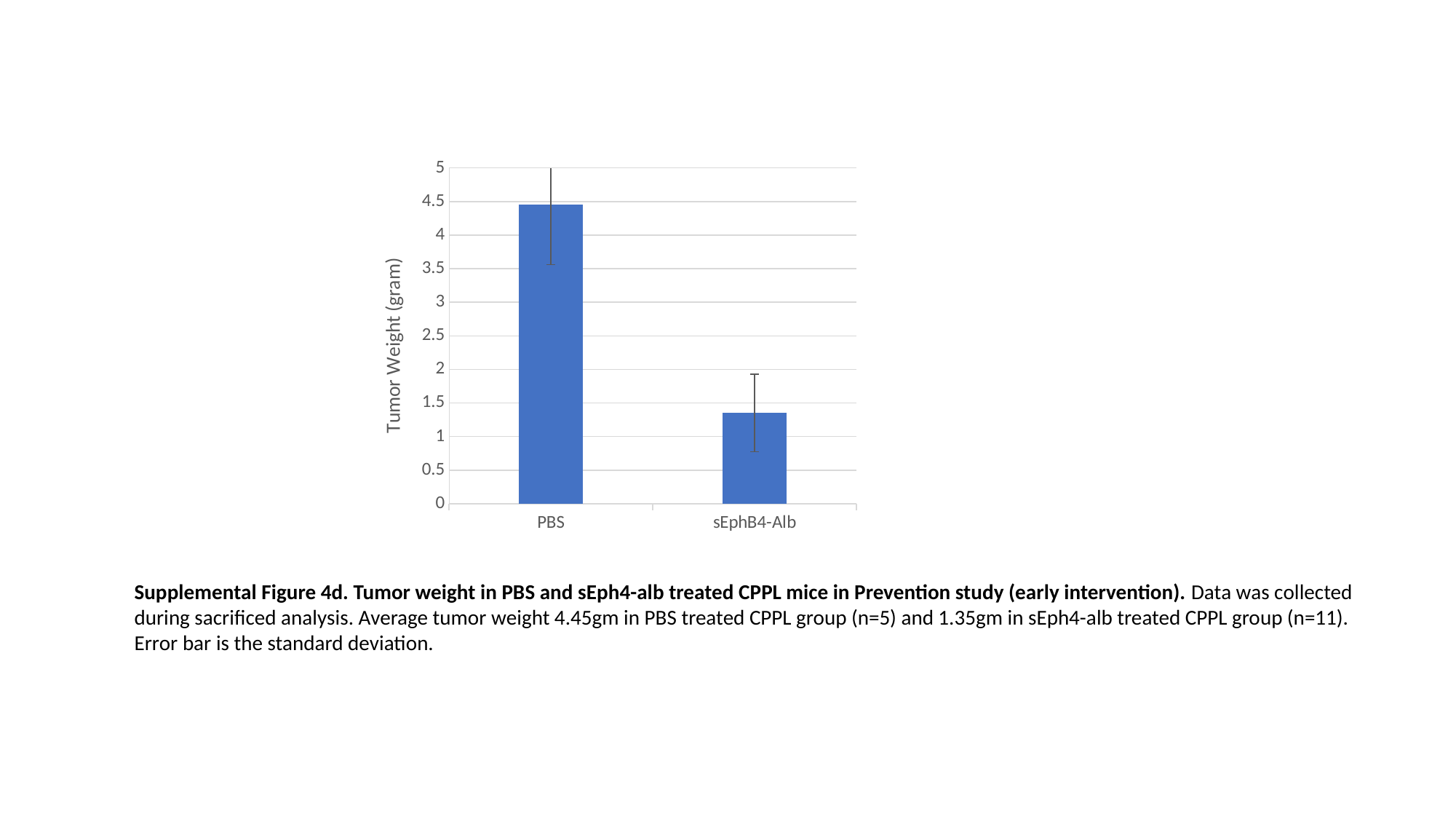

### Chart
| Category | Tumor weight |
|---|---|
| PBS | 4.45 |
| sEphB4-Alb | 1.35 |
Supplemental Figure 4d. Tumor weight in PBS and sEph4-alb treated CPPL mice in Prevention study (early intervention). Data was collected during sacrificed analysis. Average tumor weight 4.45gm in PBS treated CPPL group (n=5) and 1.35gm in sEph4-alb treated CPPL group (n=11). Error bar is the standard deviation.
| Mice # | 8 | 8 | 8 | 6 | 6 | 6 |
| --- | --- | --- | --- | --- | --- | --- |
| Mice # | 15 | 15 | 15 | 14 | 12 | 11 |
| --- | --- | --- | --- | --- | --- | --- |

## Slide 10
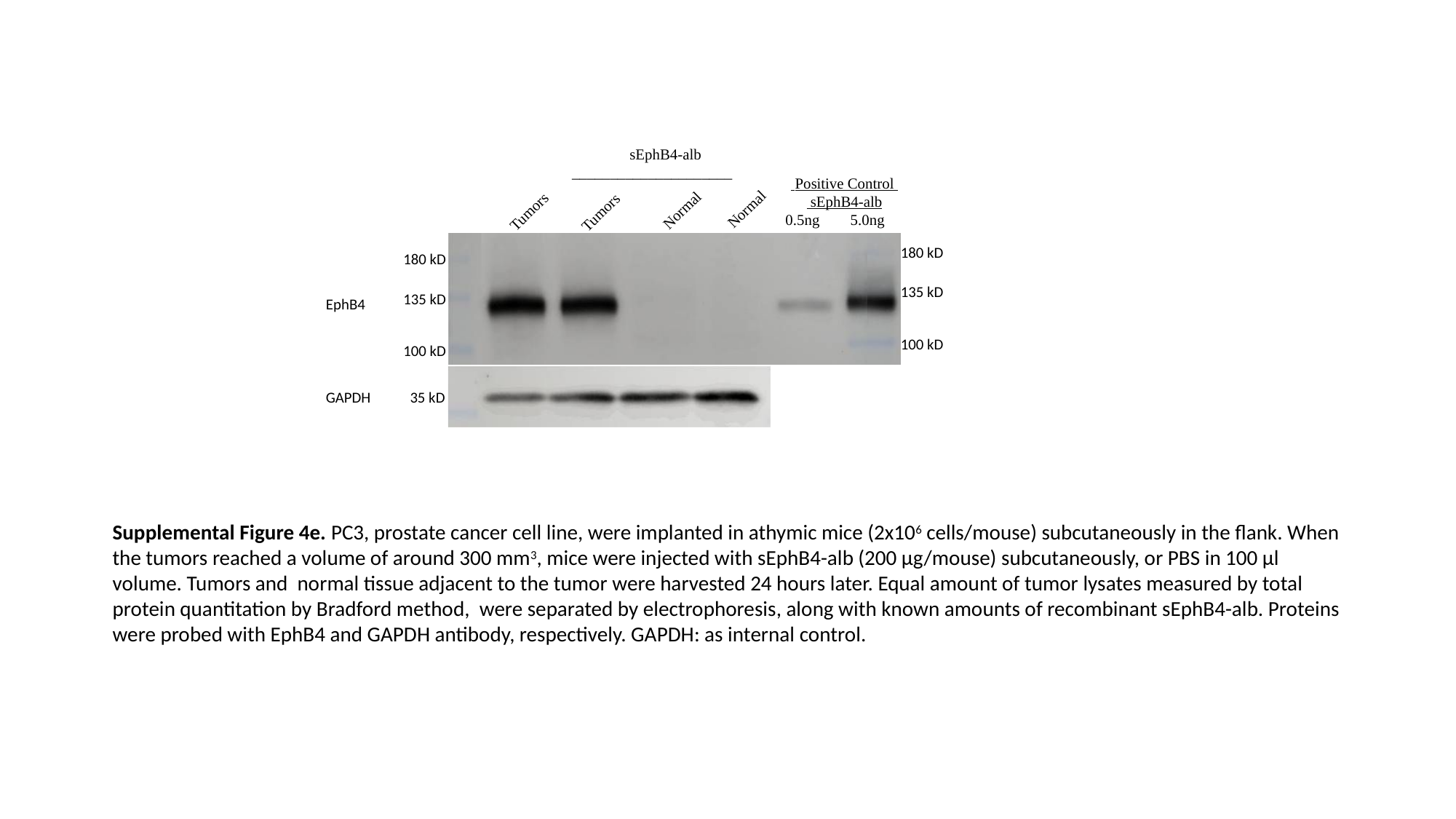

sEphB4-alb
_____________________
180 kD
180 kD
135 kD
135 kD
100 kD
100 kD
 Positive Control
 sEphB4-alb
Tumors
Tumors
Normal
Normal
 0.5ng 5.0ng
EphB4
GAPDH
35 kD
Supplemental Figure 4e. PC3, prostate cancer cell line, were implanted in athymic mice (2x10^6^ cells/mouse) subcutaneously in the flank. When the tumors reached a volume of around 300 mm3, mice were injected with sEphB4-alb (200 µg/mouse) subcutaneously, or PBS in 100 µl volume. Tumors and  normal tissue adjacent to the tumor were harvested 24 hours later. Equal amount of tumor lysates measured by total protein quantitation by Bradford method,  were separated by electrophoresis, along with known amounts of recombinant sEphB4-alb. Proteins were probed with EphB4 and GAPDH antibody, respectively. GAPDH: as internal control.

## Slide 11
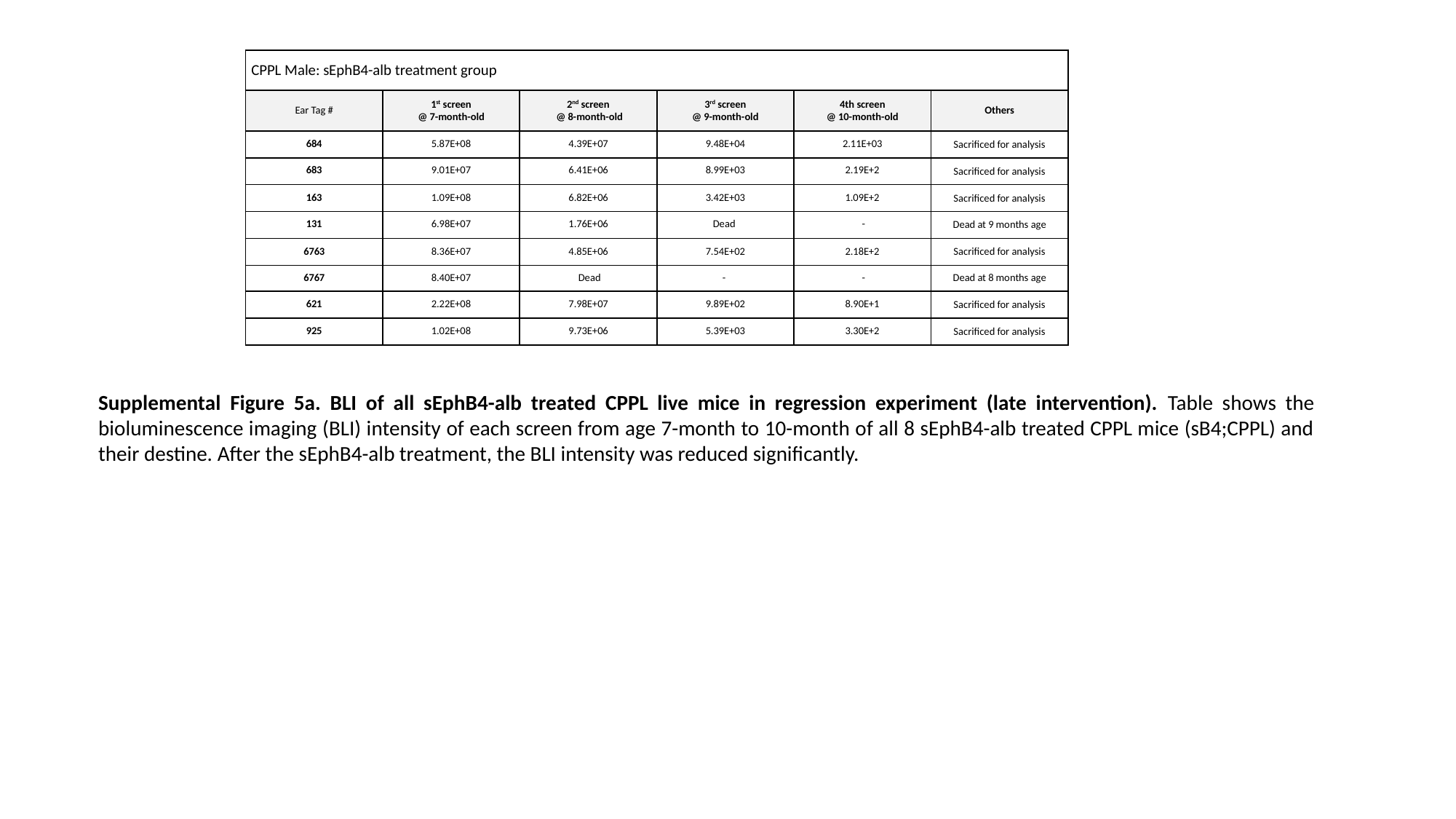

| CPPL Male: sEphB4-alb treatment group | | | | | |
| --- | --- | --- | --- | --- | --- |
| Ear Tag # | 1st screen @ 7-month-old | 2nd screen @ 8-month-old | 3rd screen @ 9-month-old | 4th screen @ 10-month-old | Others |
| 684 | 5.87E+08 | 4.39E+07 | 9.48E+04 | 2.11E+03 | Sacrificed for analysis |
| 683 | 9.01E+07 | 6.41E+06 | 8.99E+03 | 2.19E+2 | Sacrificed for analysis |
| 163 | 1.09E+08 | 6.82E+06 | 3.42E+03 | 1.09E+2 | Sacrificed for analysis |
| 131 | 6.98E+07 | 1.76E+06 | Dead | - | Dead at 9 months age |
| 6763 | 8.36E+07 | 4.85E+06 | 7.54E+02 | 2.18E+2 | Sacrificed for analysis |
| 6767 | 8.40E+07 | Dead | - | - | Dead at 8 months age |
| 621 | 2.22E+08 | 7.98E+07 | 9.89E+02 | 8.90E+1 | Sacrificed for analysis |
| 925 | 1.02E+08 | 9.73E+06 | 5.39E+03 | 3.30E+2 | Sacrificed for analysis |
Supplemental Figure 5a. BLI of all sEphB4-alb treated CPPL live mice in regression experiment (late intervention). Table shows the bioluminescence imaging (BLI) intensity of each screen from age 7-month to 10-month of all 8 sEphB4-alb treated CPPL mice (sB4;CPPL) and their destine. After the sEphB4-alb treatment, the BLI intensity was reduced significantly.

## Slide 12
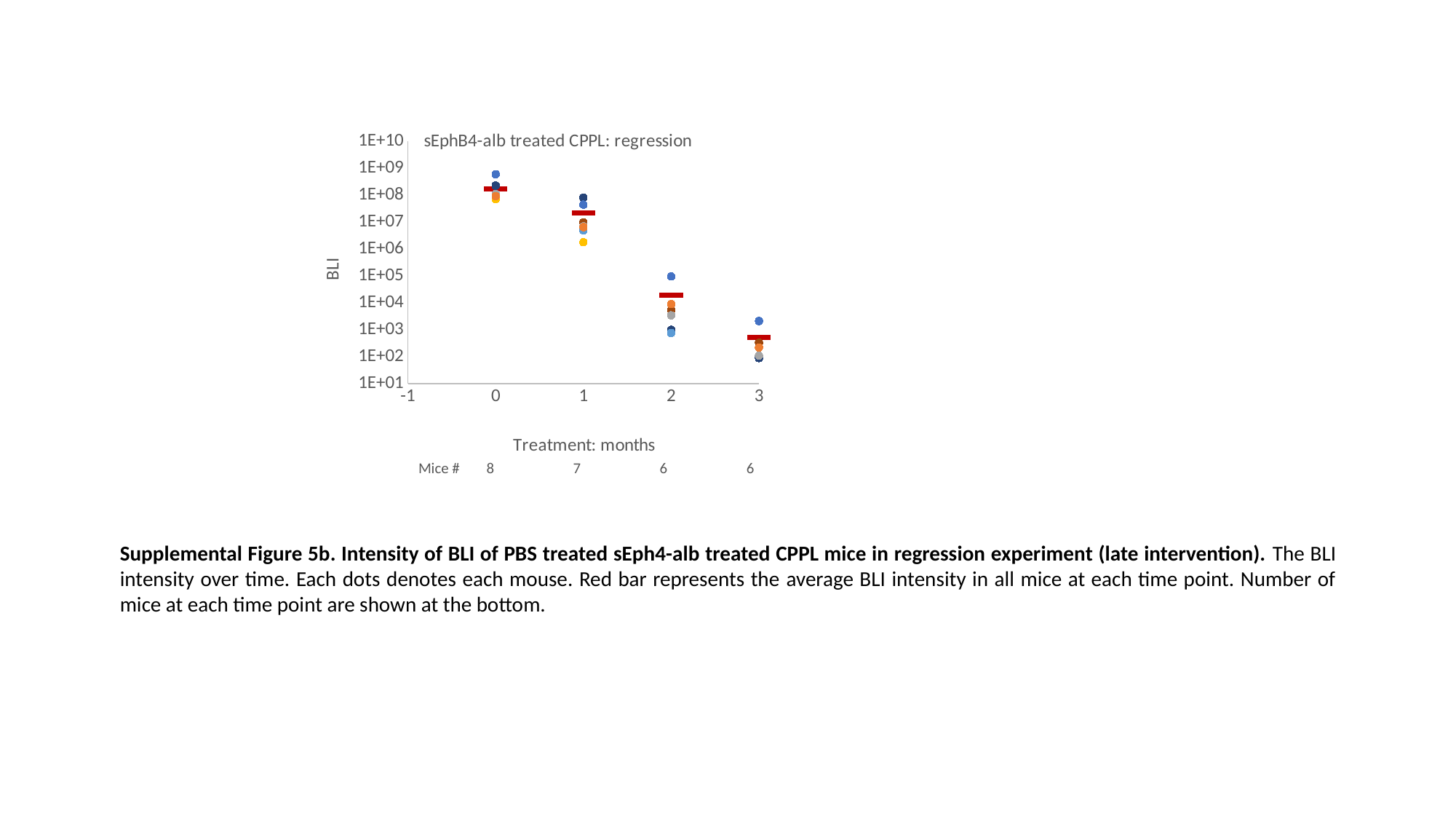

### Chart: sEphB4-alb treated CPPL: regression
| Category | 1 | 2 | 3 | 4 | 5 | 6 | 7 | 8 | AVE |
|---|---|---|---|---|---|---|---|---|---|| Mice # | 8 | 7 | 6 | 6 |
| --- | --- | --- | --- | --- |
Supplemental Figure 5b. Intensity of BLI of PBS treated sEph4-alb treated CPPL mice in regression experiment (late intervention). The BLI intensity over time. Each dots denotes each mouse. Red bar represents the average BLI intensity in all mice at each time point. Number of mice at each time point are shown at the bottom.

## Slide 13
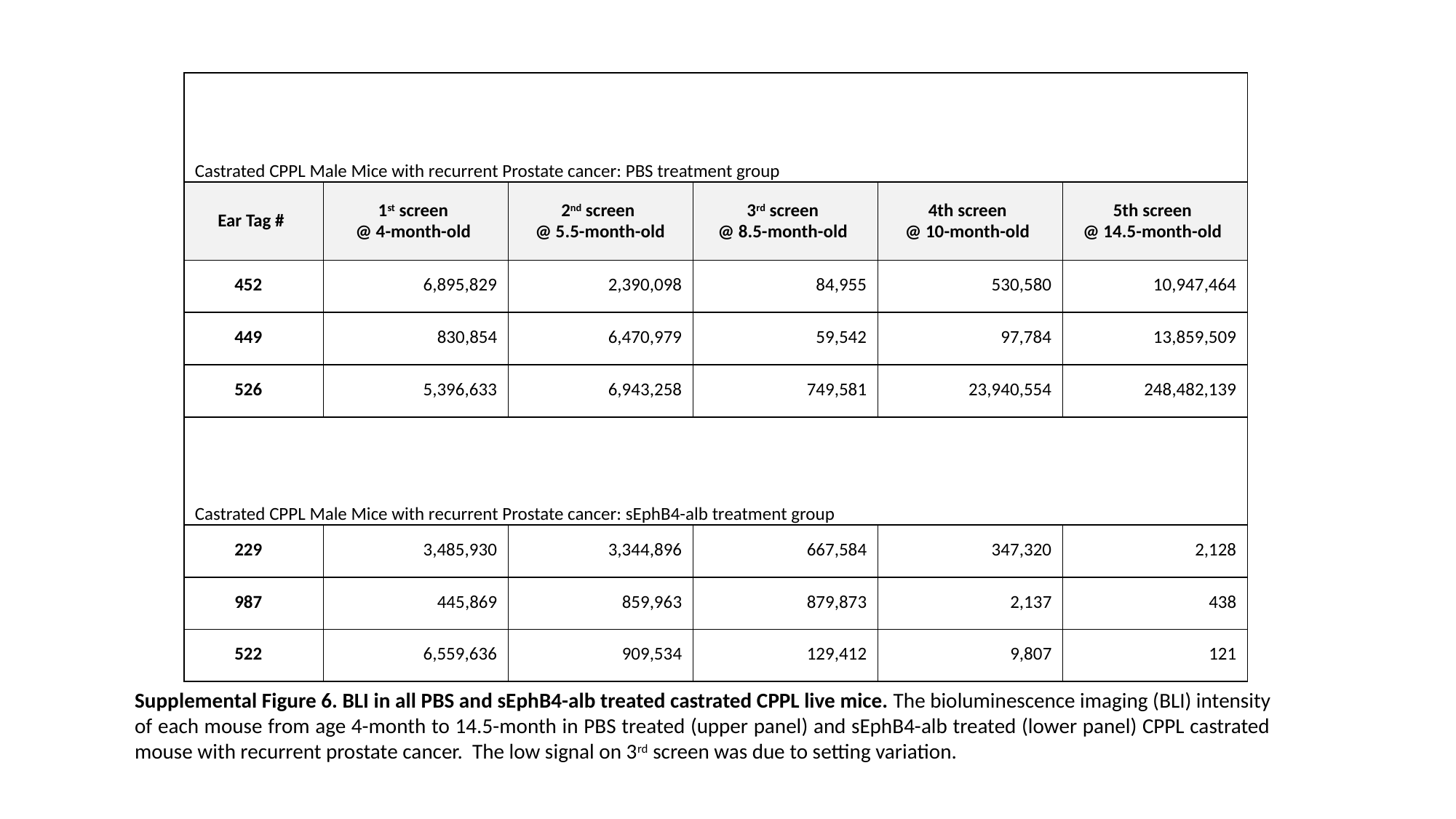

| Castrated CPPL Male Mice with recurrent Prostate cancer: PBS treatment group | | | | | |
| --- | --- | --- | --- | --- | --- |
| Ear Tag # | 1st screen @ 4-month-old | 2nd screen @ 5.5-month-old | 3rd screen @ 8.5-month-old | 4th screen @ 10-month-old | 5th screen @ 14.5-month-old |
| 452 | 6,895,829 | 2,390,098 | 84,955 | 530,580 | 10,947,464 |
| 449 | 830,854 | 6,470,979 | 59,542 | 97,784 | 13,859,509 |
| 526 | 5,396,633 | 6,943,258 | 749,581 | 23,940,554 | 248,482,139 |
| Castrated CPPL Male Mice with recurrent Prostate cancer: sEphB4-alb treatment group | | | | | |
| 229 | 3,485,930 | 3,344,896 | 667,584 | 347,320 | 2,128 |
| 987 | 445,869 | 859,963 | 879,873 | 2,137 | 438 |
| 522 | 6,559,636 | 909,534 | 129,412 | 9,807 | 121 |
Supplemental Figure 6. BLI in all PBS and sEphB4-alb treated castrated CPPL live mice. The bioluminescence imaging (BLI) intensity of each mouse from age 4-month to 14.5-month in PBS treated (upper panel) and sEphB4-alb treated (lower panel) CPPL castrated mouse with recurrent prostate cancer. The low signal on 3rd screen was due to setting variation.

## Slide 14
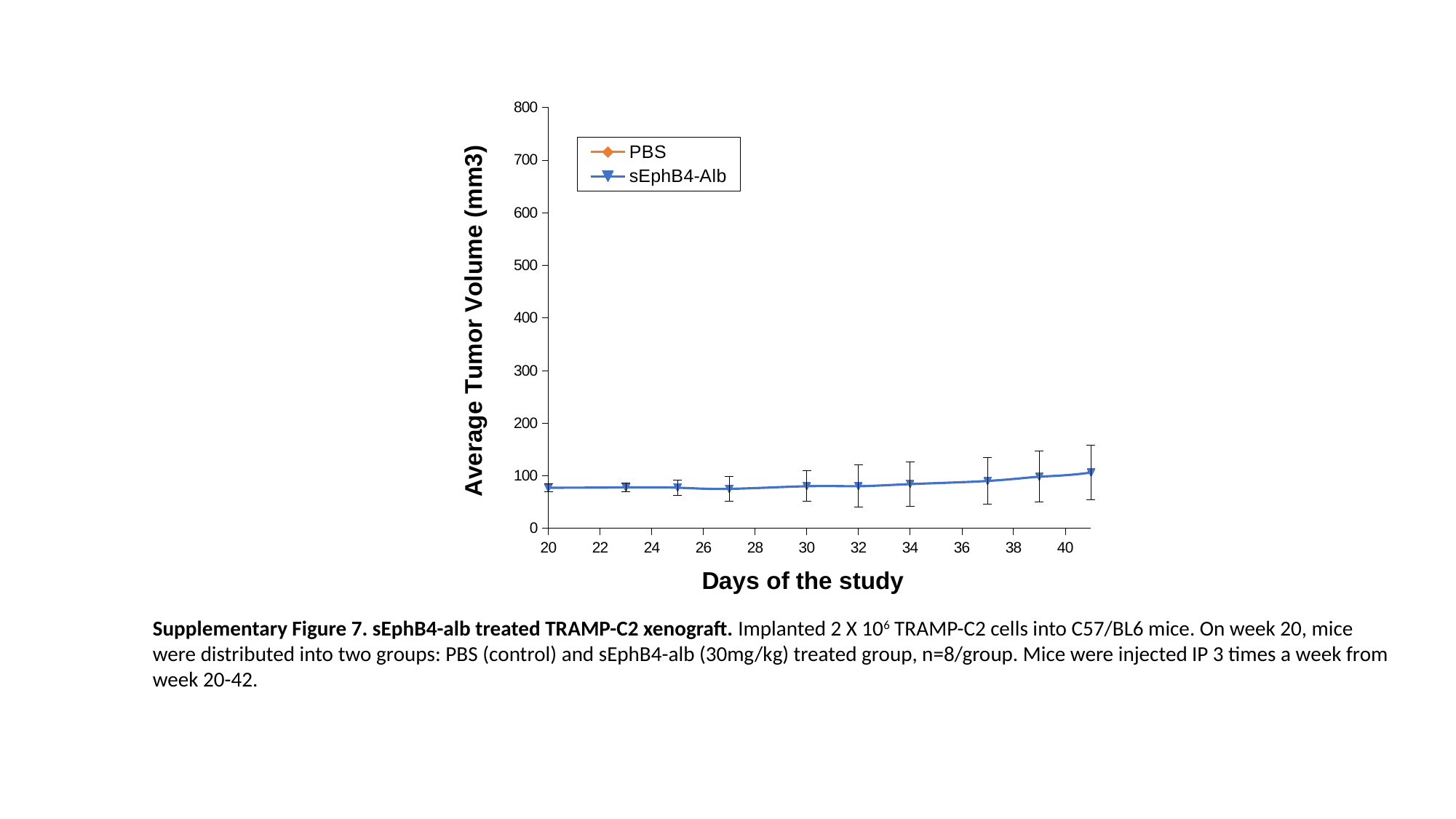

### Chart
| Category | PBS | sEphB4-Alb |
|---|---|---|Supplementary Figure 7. sEphB4-alb treated TRAMP-C2 xenograft. Implanted 2 X 106 TRAMP-C2 cells into C57/BL6 mice. On week 20, mice were distributed into two groups: PBS (control) and sEphB4-alb (30mg/kg) treated group, n=8/group. Mice were injected IP 3 times a week from week 20-42.

## Slide 15
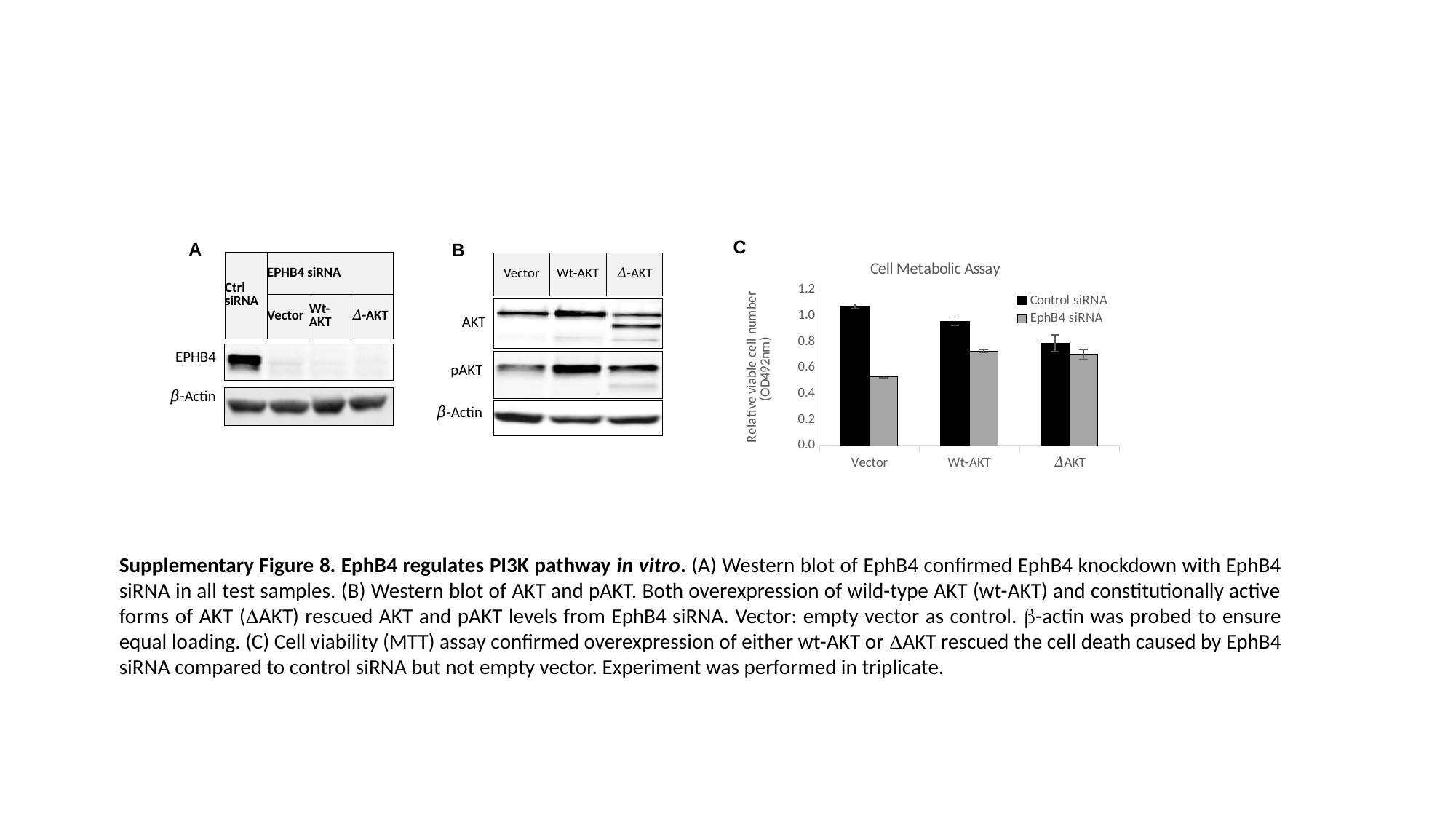

C
A
B
| Ctrl siRNA | EPHB4 siRNA | | |
| --- | --- | --- | --- |
| | Vector | Wt-AKT | 𝛥-AKT |
### Chart: Cell Metabolic Assay
| Category | Control siRNA | EphB4 siRNA |
|---|---|---|
| Vector | 1.0741499662399294 | 0.529450005292892 |
| Wt-AKT | 0.9576499998569483 | 0.7281500101089478 |
| 𝛥AKT | 0.7890500128269197 | 0.7023499906063081 || Vector | Wt-AKT | 𝛥-AKT |
| --- | --- | --- |
AKT
EPHB4
pAKT
𝛽-Actin
𝛽-Actin
Supplementary Figure 8. EphB4 regulates PI3K pathway in vitro. (A) Western blot of EphB4 confirmed EphB4 knockdown with EphB4 siRNA in all test samples. (B) Western blot of AKT and pAKT. Both overexpression of wild-type AKT (wt-AKT) and constitutionally active forms of AKT (ΔAKT) rescued AKT and pAKT levels from EphB4 siRNA. Vector: empty vector as control. β-actin was probed to ensure equal loading. (C) Cell viability (MTT) assay confirmed overexpression of either wt-AKT or ΔAKT rescued the cell death caused by EphB4 siRNA compared to control siRNA but not empty vector. Experiment was performed in triplicate.

## Slide 16
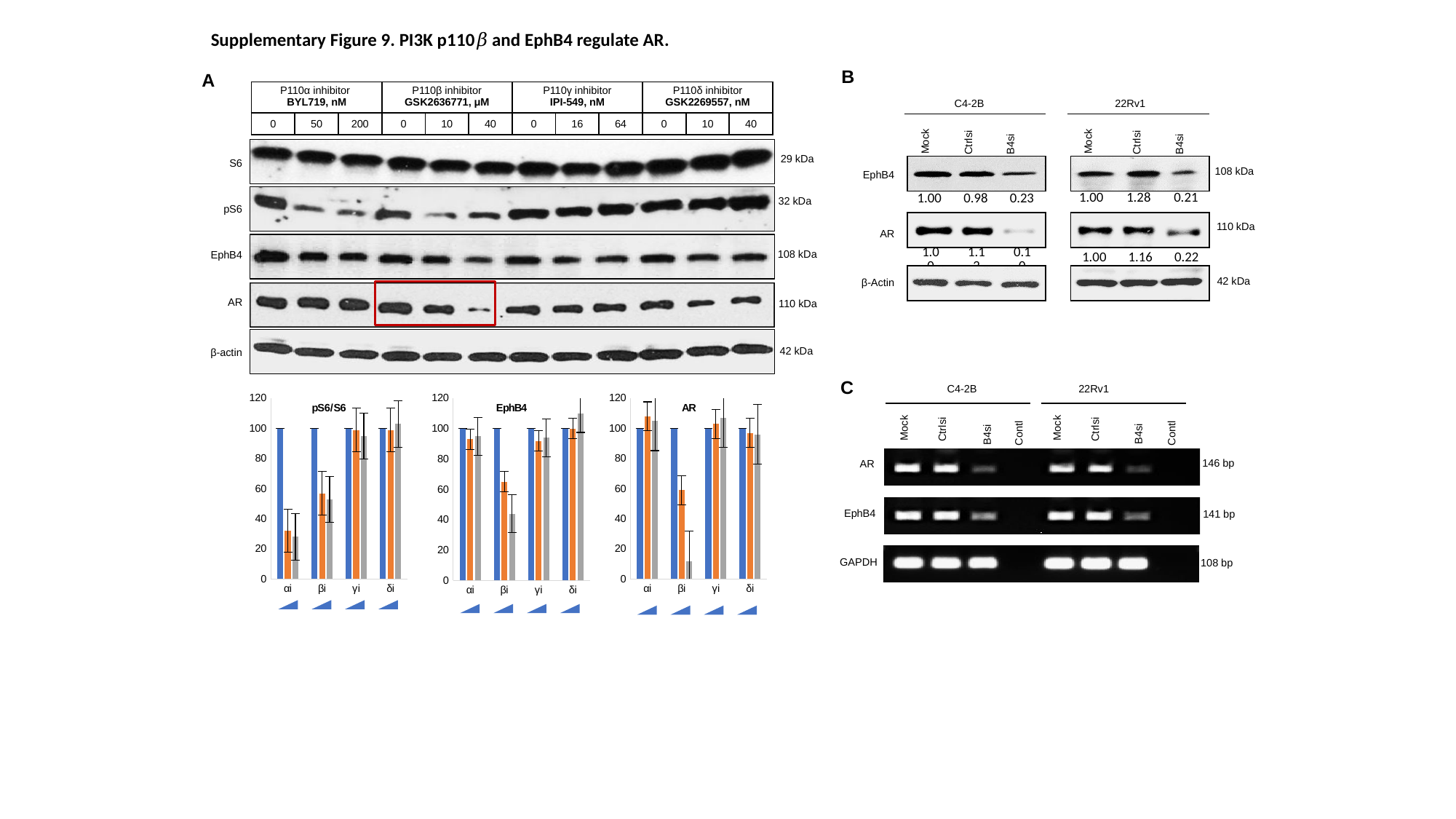

Supplementary Figure 9. PI3K p110𝛽 and EphB4 regulate AR.
B
A
| P110α inhibitor BYL719, nM | | | P110β inhibitor GSK2636771, μM | | | P110γ inhibitor IPI-549, nM | | | P110δ inhibitor GSK2269557, nM | | |
| --- | --- | --- | --- | --- | --- | --- | --- | --- | --- | --- | --- |
| 0 | 50 | 200 | 0 | 10 | 40 | 0 | 16 | 64 | 0 | 10 | 40 |
C4-2B
22Rv1
Ctrlsi
Ctrlsi
B4si
B4si
Mock
Mock
29 kDa
S6
108 kDa
EphB4
| 1.00 | 1.28 | 0.21 |
| --- | --- | --- |
| 1.00 | 0.98 | 0.23 |
| --- | --- | --- |
32 kDa
pS6
110 kDa
AR
| 1.00 | 1.12 | 0.10 |
| --- | --- | --- |
| 1.00 | 1.16 | 0.22 |
| --- | --- | --- |
108 kDa
EphB4
42 kDa
β-Actin
AR
110 kDa
42 kDa
β-actin
C
C4-2B
22Rv1
### Chart: pS6/S6
| Category | Series 1 | Series 2 | Series 3 |
|---|---|---|---|
| αi | 100.0 | 32.0 | 28.0 |
| βi | 100.0 | 57.0 | 53.0 |
| γi | 100.0 | 99.0 | 95.0 |
| δi | 100.0 | 99.0 | 103.0 |
### Chart: EphB4
| Category | Series 1 | Series 2 | Series 3 |
|---|---|---|---|
| αi | 100.0 | 93.0 | 95.0 |
| βi | 100.0 | 65.0 | 44.0 |
| γi | 100.0 | 92.0 | 94.0 |
| δi | 100.0 | 100.0 | 110.0 |
### Chart: AR
| Category | Series 1 | Series 2 | Series 3 |
|---|---|---|---|
| αi | 100.0 | 108.0 | 105.0 |
| βi | 100.0 | 59.0 | 12.0 |
| γi | 100.0 | 103.0 | 107.0 |
| δi | 100.0 | 97.0 | 96.0 |Mock
Mock
B4si
Ctrlsi
Ctrlsi
B4si
Contl
Contl
146 bp
AR
EphB4
141 bp
GAPDH
108 bp

## Slide 17
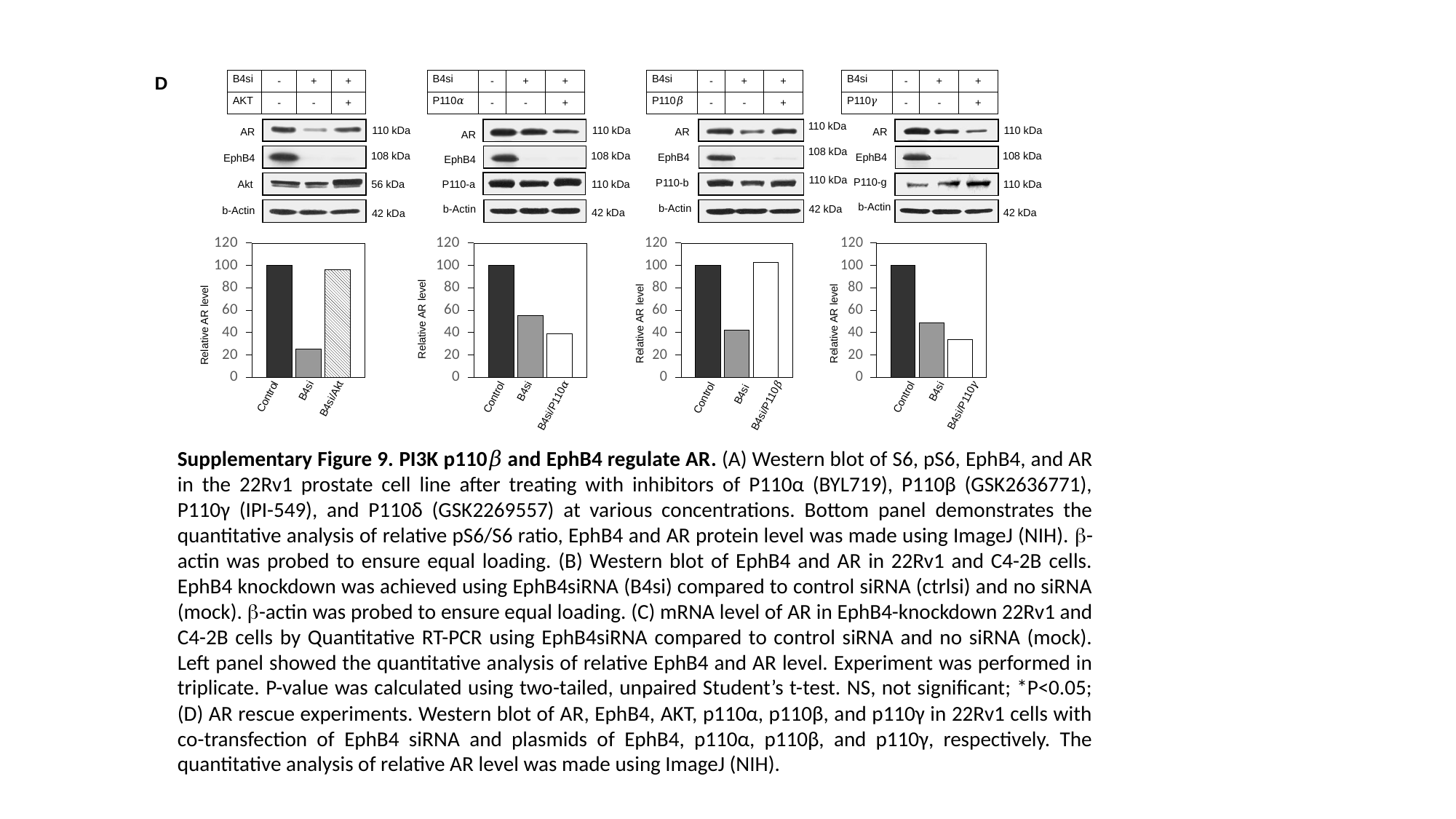

D
| B4si | - | + | + |
| --- | --- | --- | --- |
| AKT | - | - | + |
| B4si | - | + | + |
| --- | --- | --- | --- |
| P110𝛼 | - | - | + |
| B4si | - | + | + |
| --- | --- | --- | --- |
| P110𝛽 | - | - | + |
| B4si | - | + | + |
| --- | --- | --- | --- |
| P110𝛾 | - | - | + |
110 kDa
110 kDa
110 kDa
110 kDa
AR
AR
AR
AR
108 kDa
108 kDa
108 kDa
108 kDa
EphB4
EphB4
EphB4
EphB4
110 kDa
P110-g
P110-b
110 kDa
110 kDa
P110-a
Akt
56 kDa
b-Actin
b-Actin
42 kDa
b-Actin
b-Actin
42 kDa
42 kDa
42 kDa
### Chart
| Category | Control | siEphB4 | Akt+siEphB4 |
|---|---|---|---|
### Chart
| Category | Connrol | siEphB4 | p110𝝰+siEphB4 |
|---|---|---|---|
### Chart
| Category | Connrol | siEphB4 | p110𝝱+siEphB4 |
|---|---|---|---|
### Chart
| Category | Connrol | siEphB4 | p110𝝲+siEphB4 |
|---|---|---|---|Relative AR level
Relative AR level
Relative AR level
Relative AR level
B4si
B4si
B4si
B4si
Control
Control
Control
Control
B4si/Akt
B4si/P110𝛾
B4si/P110𝛼
B4si/P110𝛽
Supplementary Figure 9. PI3K p110𝛽 and EphB4 regulate AR. (A) Western blot of S6, pS6, EphB4, and AR in the 22Rv1 prostate cell line after treating with inhibitors of P110α (BYL719), P110β (GSK2636771), P110γ (IPI-549), and P110δ (GSK2269557) at various concentrations. Bottom panel demonstrates the quantitative analysis of relative pS6/S6 ratio, EphB4 and AR protein level was made using ImageJ (NIH). β-actin was probed to ensure equal loading. (B) Western blot of EphB4 and AR in 22Rv1 and C4-2B cells. EphB4 knockdown was achieved using EphB4siRNA (B4si) compared to control siRNA (ctrlsi) and no siRNA (mock). β-actin was probed to ensure equal loading. (C) mRNA level of AR in EphB4-knockdown 22Rv1 and C4-2B cells by Quantitative RT-PCR using EphB4siRNA compared to control siRNA and no siRNA (mock). Left panel showed the quantitative analysis of relative EphB4 and AR level. Experiment was performed in triplicate. P-value was calculated using two-tailed, unpaired Student’s t-test. NS, not significant; *P<0.05; (D) AR rescue experiments. Western blot of AR, EphB4, AKT, p110α, p110β, and p110γ in 22Rv1 cells with co-transfection of EphB4 siRNA and plasmids of EphB4, p110α, p110β, and p110γ, respectively. The quantitative analysis of relative AR level was made using ImageJ (NIH).

## Slide 18
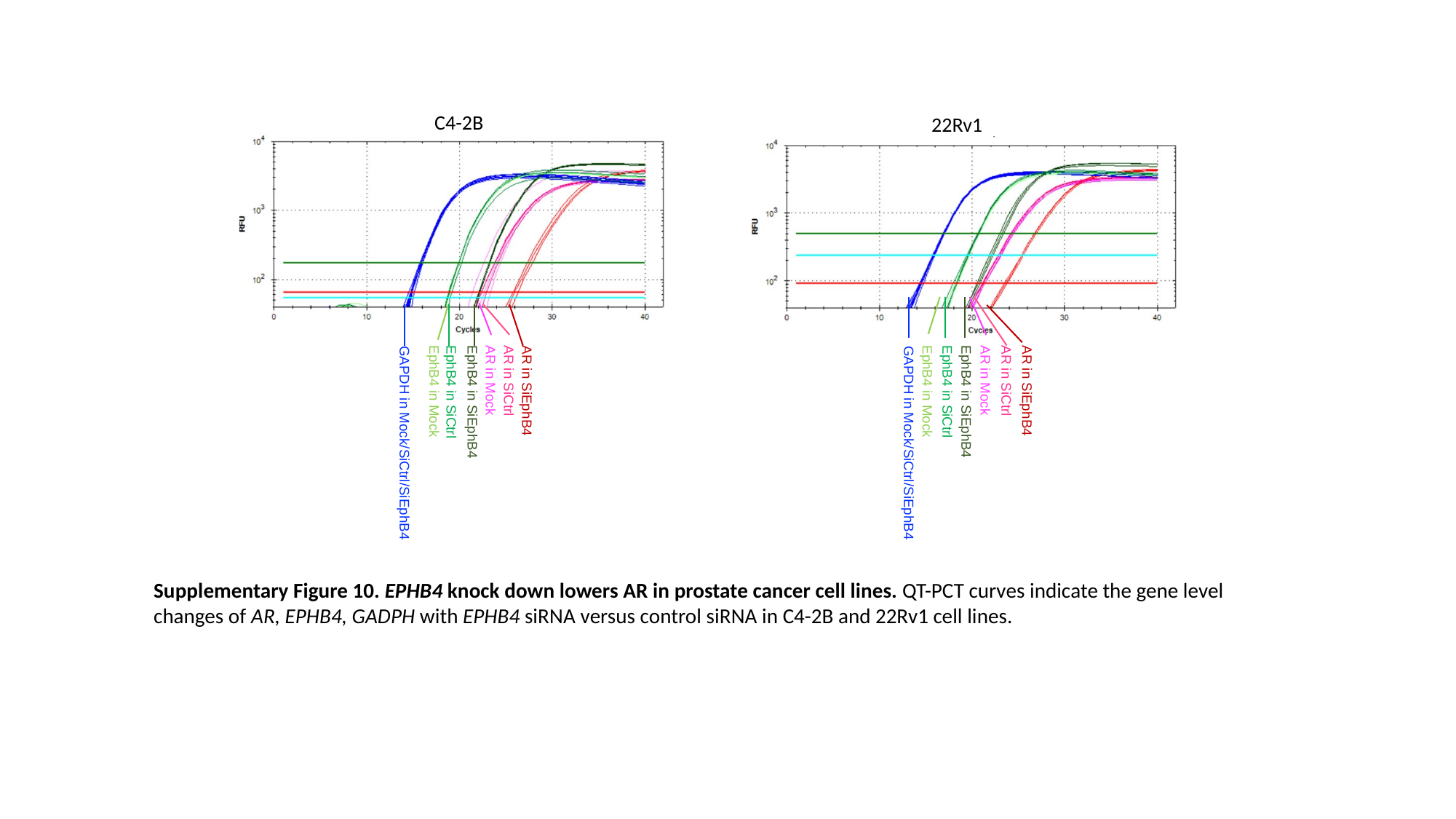

C4-2B
22Rv1
AR in Mock
AR in Mock
AR in SiCtrl
AR in SiCtrl
AR in SiEphB4
AR in SiEphB4
EphB4 in Mock
EphB4 in Mock
EphB4 in SiCtrl
EphB4 in SiCtrl
EphB4 in SiEphB4
EphB4 in SiEphB4
GAPDH in Mock/SiCtrl/SiEphB4
GAPDH in Mock/SiCtrl/SiEphB4
Supplementary Figure 10. EPHB4 knock down lowers AR in prostate cancer cell lines. QT-PCT curves indicate the gene level changes of AR, EPHB4, GADPH with EPHB4 siRNA versus control siRNA in C4-2B and 22Rv1 cell lines.

## Slide 19
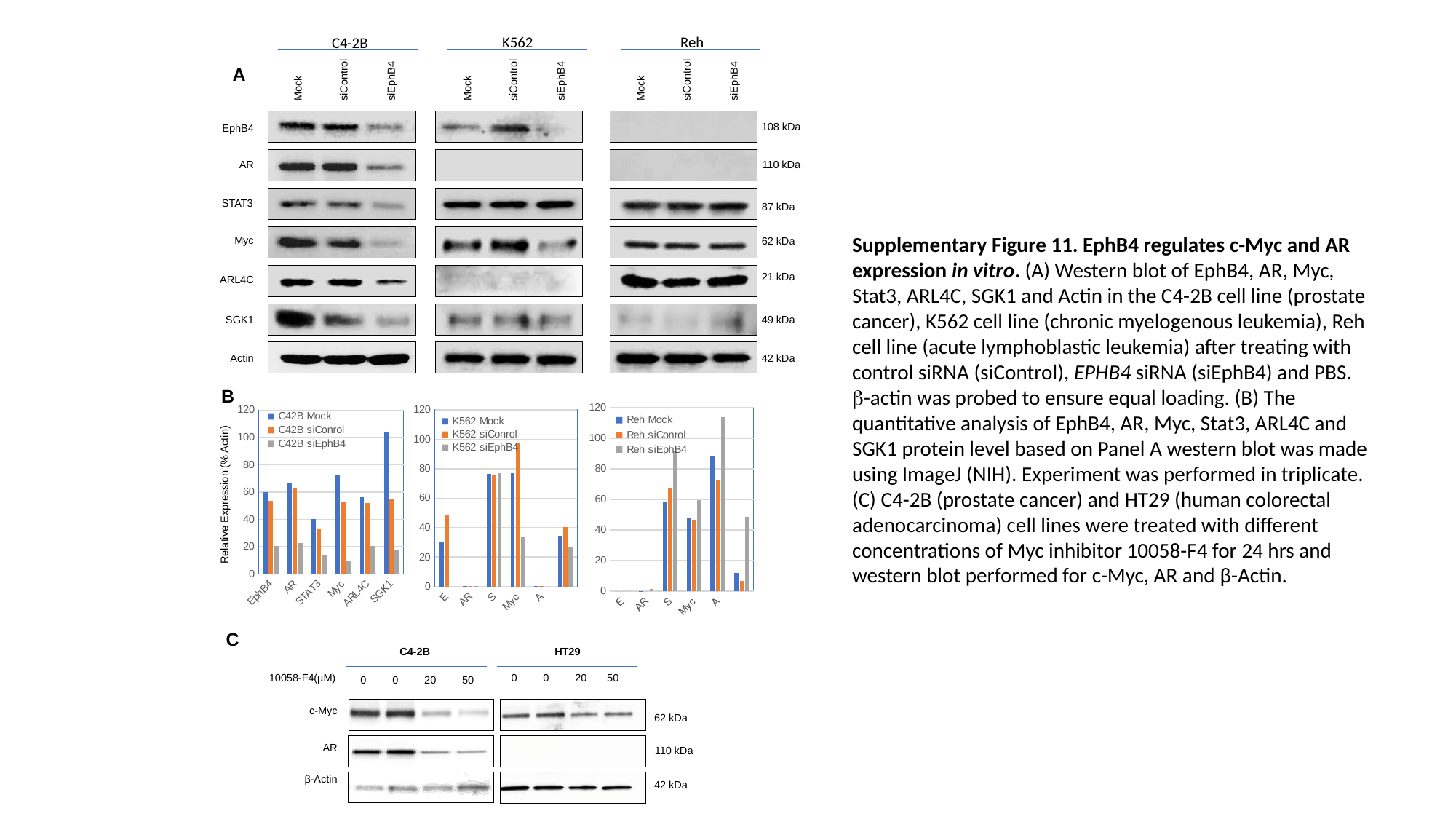

K562
Reh
C4-2B
A
siControl
siControl
siControl
siEphB4
siEphB4
siEphB4
Mock
Mock
Mock
108 kDa
EphB4
110 kDa
AR
STAT3
87 kDa
Supplementary Figure 11. EphB4 regulates c-Myc and AR expression in vitro. (A) Western blot of EphB4, AR, Myc, Stat3, ARL4C, SGK1 and Actin in the C4-2B cell line (prostate cancer), K562 cell line (chronic myelogenous leukemia), Reh cell line (acute lymphoblastic leukemia) after treating with control siRNA (siControl), EPHB4 siRNA (siEphB4) and PBS. β-actin was probed to ensure equal loading. (B) The quantitative analysis of EphB4, AR, Myc, Stat3, ARL4C and SGK1 protein level based on Panel A western blot was made using ImageJ (NIH). Experiment was performed in triplicate. (C) C4-2B (prostate cancer) and HT29 (human colorectal adenocarcinoma) cell lines were treated with different concentrations of Myc inhibitor 10058-F4 for 24 hrs and western blot performed for c-Myc, AR and β-Actin.
Myc
62 kDa
21 kDa
ARL4C
SGK1
49 kDa
42 kDa
Actin
B
### Chart
| Category | Reh | Reh | Reh |
|---|---|---|---|
| EphB4 | 0.0 | 0.0 | 0.0 |
| AR | 0.2282517666248844 | 0.0 | 1.671364216713642 |
| STAT3 | 58.0761167641891 | 67.16040967394197 | 91.60174411601744 |
| Myc | 47.587117915482985 | 46.9225628938344 | 59.72802389728024 |
| ARL4C | 87.90393355702372 | 72.4031102745903 | 113.88017633880176 |
| SGK1 | 12.200248504321458 | 6.723355205842201 | 48.76803738768037 |
### Chart
| Category | K562 | K562 | K562 |
|---|---|---|---|
| EphB4 | 30.372504702351506 | 48.70030150921301 | 0.0 |
| AR | 0.10134847966382819 | 0.09689135041538259 | 0.0939859122191792 |
| STAT3 | 76.67938789877357 | 75.62109439301216 | 76.68320683974896 |
| Myc | 76.99956191302338 | 97.37705737843261 | 33.39612535497367 |
| ARL4C | 0.07650175561721224 | 0.4438248954511074 | 0.0 |
| SGK1 | 34.379583839167594 | 40.40619354516075 | 27.082495376499477 |
### Chart
| Category | C42B | C42B | C42B |
|---|---|---|---|
| EphB4 | 59.75822246977322 | 53.693839640199045 | 20.42924480994885 |
| AR | 66.0967293341224 | 62.63266619373123 | 22.68212737573885 |
| STAT3 | 40.30278989576596 | 32.683538551464686 | 13.491187937081028 |
| Myc | 72.64429058009385 | 53.08451610553282 | 9.445964726277634 |
| ARL4C | 56.06614986322603 | 51.845402148329434 | 20.452646006752918 |
| SGK1 | 103.67668209992469 | 55.02557493742856 | 17.52240226816498 |Relative Expression (% Actin)
C
C4-2B
HT29
10058-F4(µM)
0 0 20 50
0 0 20 50
c-Myc
62 kDa
AR
110 kDa
β-Actin
42 kDa

## Slide 20
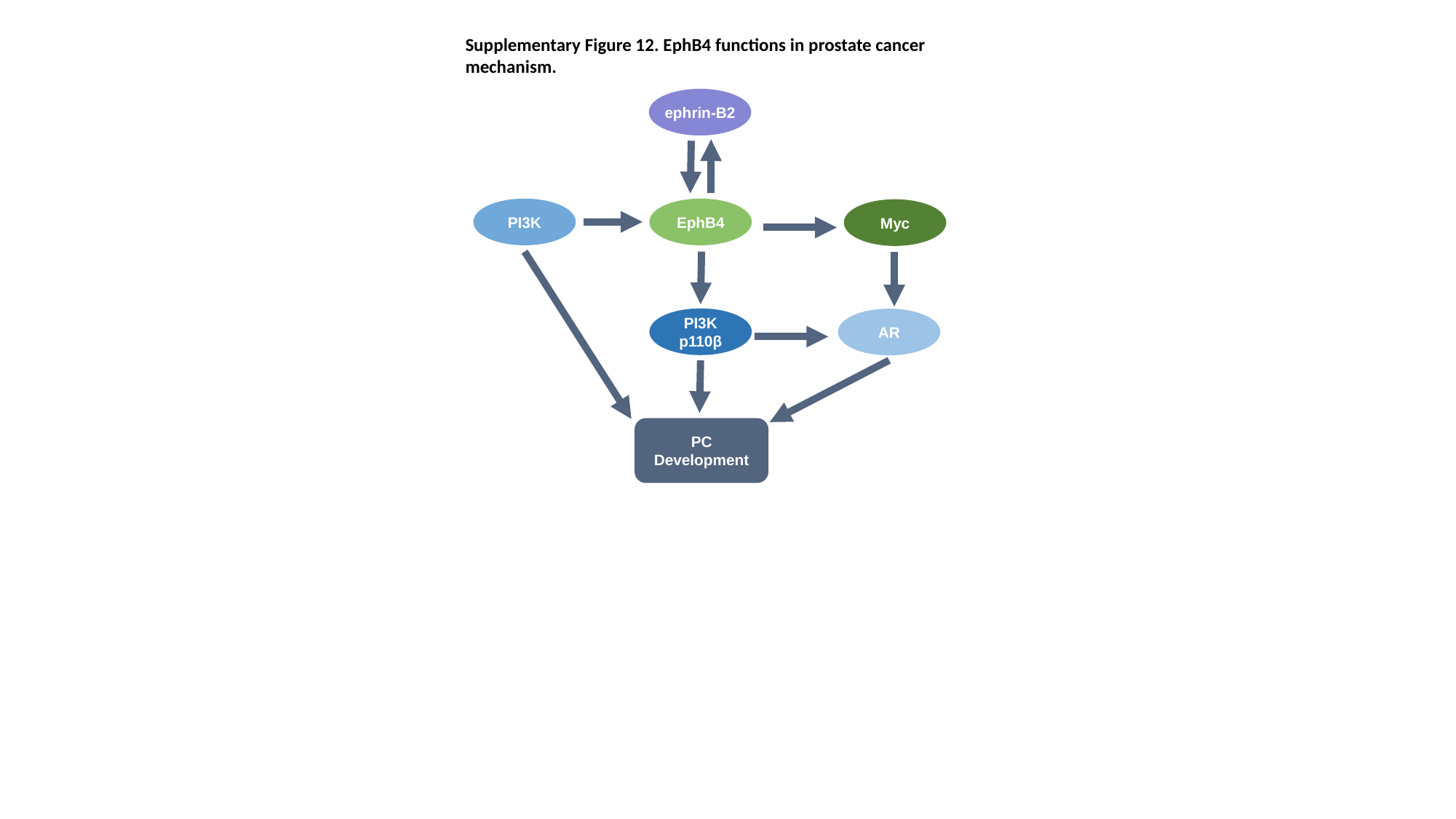

Supplementary Figure 12. EphB4 functions in prostate cancer mechanism.
ephrin-B2
PI3K
EphB4
Myc
PI3K p110β
AR
PC Development

## Slide 21
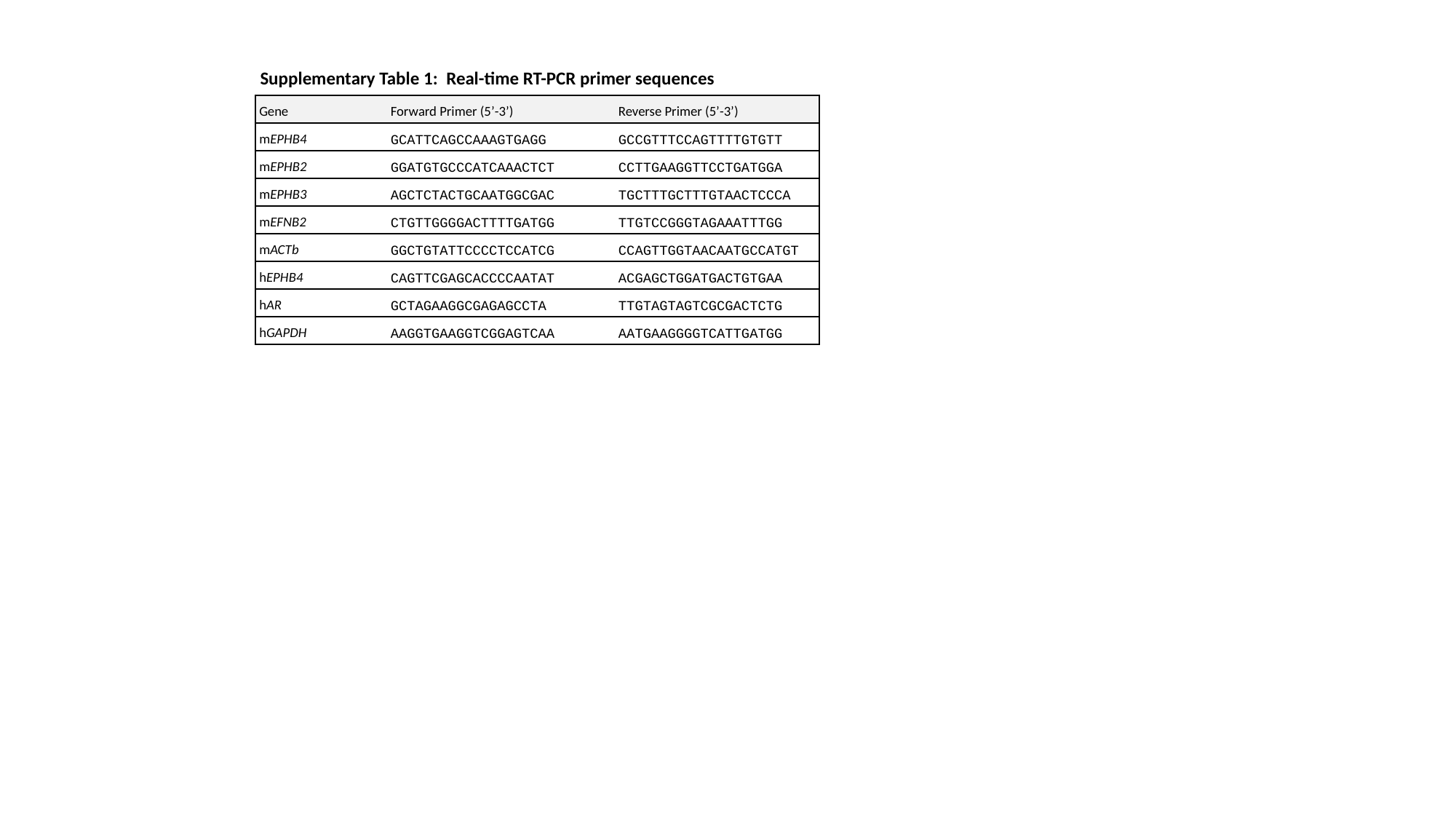

Supplementary Table 1: Real-time RT-PCR primer sequences
| Gene | Forward Primer (5’-3’) | Reverse Primer (5’-3’) |
| --- | --- | --- |
| mEPHB4 | GCATTCAGCCAAAGTGAGG | GCCGTTTCCAGTTTTGTGTT |
| mEPHB2 | GGATGTGCCCATCAAACTCT | CCTTGAAGGTTCCTGATGGA |
| mEPHB3 | AGCTCTACTGCAATGGCGAC | TGCTTTGCTTTGTAACTCCCA |
| mEFNB2 | CTGTTGGGGACTTTTGATGG | TTGTCCGGGTAGAAATTTGG |
| mACTb | GGCTGTATTCCCCTCCATCG | CCAGTTGGTAACAATGCCATGT |
| hEPHB4 | CAGTTCGAGCACCCCAATAT | ACGAGCTGGATGACTGTGAA |
| hAR | GCTAGAAGGCGAGAGCCTA | TTGTAGTAGTCGCGACTCTG |
| hGAPDH | AAGGTGAAGGTCGGAGTCAA | AATGAAGGGGTCATTGATGG |

## Slide 22
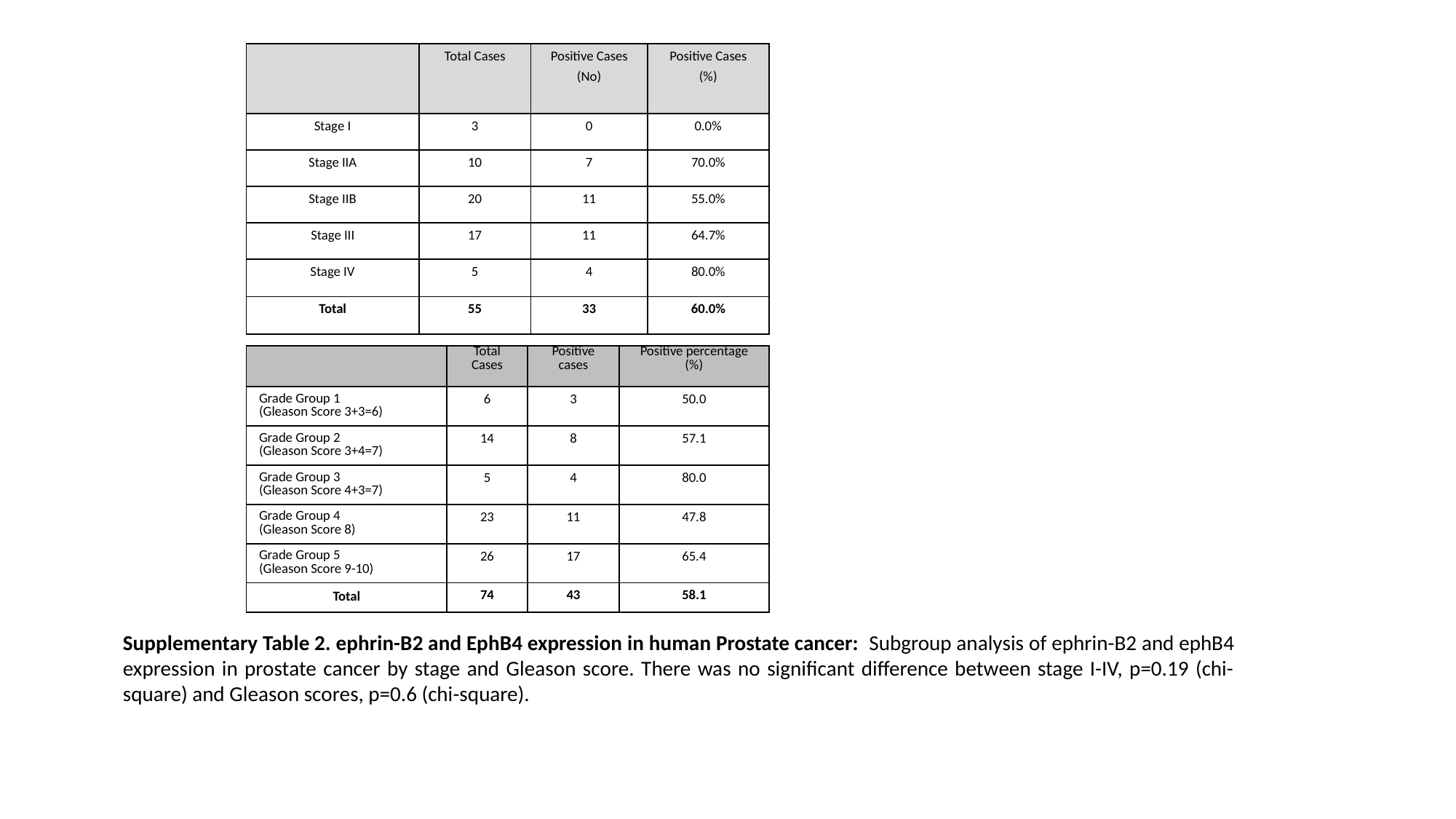

| | Total Cases | Positive Cases (No) | Positive Cases (%) |
| --- | --- | --- | --- |
| Stage I | 3 | 0 | 0.0% |
| Stage IIA | 10 | 7 | 70.0% |
| Stage IIB | 20 | 11 | 55.0% |
| Stage III | 17 | 11 | 64.7% |
| Stage IV | 5 | 4 | 80.0% |
| Total | 55 | 33 | 60.0% |
| | Total Cases | Positive cases | Positive percentage (%) |
| --- | --- | --- | --- |
| Grade Group 1 (Gleason Score 3+3=6) | 6 | 3 | 50.0 |
| Grade Group 2 (Gleason Score 3+4=7) | 14 | 8 | 57.1 |
| Grade Group 3 (Gleason Score 4+3=7) | 5 | 4 | 80.0 |
| Grade Group 4 (Gleason Score 8) | 23 | 11 | 47.8 |
| Grade Group 5 (Gleason Score 9-10) | 26 | 17 | 65.4 |
| Total | 74 | 43 | 58.1 |
Supplementary Table 2. ephrin-B2 and EphB4 expression in human Prostate cancer: Subgroup analysis of ephrin-B2 and ephB4 expression in prostate cancer by stage and Gleason score. There was no significant difference between stage I-IV, p=0.19 (chi-square) and Gleason scores, p=0.6 (chi-square).
